# Emerging hiPSC Models for Drug Discovery in Neurodegenerative Diseases

**DOI:** 10.3390/ijms22158196

**Published:** 2021-07-30

**Authors:** Dorit Trudler, Swagata Ghatak, Stuart A. Lipton

**Affiliations:** 1 Neurodegeneration New Medicines Center and Department of Molecular Medicine, The Scripps Research Institute, La Jolla, CA 92037, USA; dtrudler@scripps.edu (D.T.); swagata@scripps.edu (S.G.); 2 Department of Neurosciences, University of California San Diego School of Medicine, La Jolla, CA 92093, USA

**Keywords:** neurodegeneration, Alzheimer’s disease

## Abstract

Neurodegenerative diseases affect millions of people worldwide and are characterized by the chronic and progressive deterioration of neural function. Neurodegenerative diseases, such as Alzheimer’s disease (AD), Parkinson’s disease (PD), amyotrophic lateral sclerosis (ALS), and Huntington’s disease (HD), represent a huge social and economic burden due to increasing prevalence in our aging society, severity of symptoms, and lack of effective disease-modifying therapies. This lack of effective treatments is partly due to a lack of reliable models. Modeling neurodegenerative diseases is difficult because of poor access to human samples (restricted in general to postmortem tissue) and limited knowledge of disease mechanisms in a human context. Animal models play an instrumental role in understanding these diseases but fail to comprehensively represent the full extent of disease due to critical differences between humans and other mammals. The advent of human-induced pluripotent stem cell (hiPSC) technology presents an advantageous system that complements animal models of neurodegenerative diseases. Coupled with advances in gene-editing technologies, hiPSC-derived neural cells from patients and healthy donors now allow disease modeling using human samples that can be used for drug discovery.

## 1. Introduction

Neurodegenerative disorders such as Alzheimer’s disease (AD), Parkinson’s disease (PD), amyotrophic lateral sclerosis (ALS), and Huntington’s disease (HD) represent a huge social and economic burden due to their high incidence, the severity of their symptoms, and the lack of effective disease-modifying therapies. Current knowledge of the exact mechanisms of these diseases is limited. Moreover, the complexity of these diseases contributes to the lack of tractable model systems that reliably recapitulate the disease phenotypes, making the development of effective, disease-modifying treatments very difficult. Traditional approaches to studying disease, such as the use of human tissue, is restricted almost entirely to postmortem brain for neurodegenerative diseases. Animal models, though widely used, fail to comprehensively represent the full panoply of symptoms due, in part, to disease-critical differences with human physiology. Measurements of subtle changes in cognition and behavior are also difficult in animals. Although no model system is perfect, the advent of human-induced pluripotent stem cell (hiPSC) technology [1] has presented an opportunity to complement the existing assemblage of neurodegenerative disease models. Coupled with advances in gene-editing technologies, hiPSC-derived neural cells from patients and healthy donors have created new methods of modeling neurological diseases in a human context that is just beginning to be exploited for therapeutic purposes.

hiPSCs are produced by reprogramming human cells, obtained from tissues such as skin or blood, into a pluripotent state, and then differentiated into the desired cell type, e.g., neurons, astrocytes, microglia, or oligodendrocytes. This is achieved by first introducing fate-determining “pluripotency factors” into the human skin or blood cell. The original hiPSC conversion method was developed by Yamanaka et al. [1] and introduced the master transcription factors OCT4, SOX2, KLF4, and c-MYC into human fibroblasts. Methods with higher efficiency and yield were subsequently developed [2,3,4,5,6,7,8,9]. Importantly, the newer methods do not use viruses or other approaches that result in the integration of DNA sequences into the cell’s genome, which has the potential for the aberrant modulation of genes near the integration sites. While direct conversion (avoiding an intermediate pluripotent state) of cell types, e.g., fibroblasts to neurons, is also possible and may retain more epigenetic features [10], this technique does not afford a limitless supply of converted cells the way the hiPSC platform can by simply expanding the stem cell pool.

## 2. Methods for Disease Modeling

The brain is composed of various cell types, as well as many regions that differ in their functional characteristics. Over the last two decades, many differentiation protocols have been developed for the various cell types of the brain, namely neurons, astrocytes, microglia, oligodendrocytes, and blood endothelial cells of the blood–brain barrier. Different methods and modeling strategies determine which cell types are produced, which experimental questions can be addressed, and how compounds can be screened for drug development. In this section, we will describe the different strategies for differentiation of pluripotent neural precursors into terminally differentiated neural cell types.

### 2.1. 2-Dimentional (2D) Models

The most simplified approach to modeling a disease is by using a cell culture generated by a single differentiation method. The differentiation method is chosen based on the cells affected by the disease. For example, to study the interplay between excitatory and inhibitory cerebrocortical neurons, the dual-SMAD [11] inhibition protocol was developed. In this protocol, hiPSCs are exposed to patterning signals similar to those during embryonic development. Inhibiting bone morphogenetic protein (BMP) and the activin/transforming growth factor (TGF)-β pathways is a very well-established way of generating neural precursor cells (NPCs) from hiPSCs. Then, NPCs are terminally differentiated to neurons in culture conditions that contain brain-derived neurotrophic factor (BDNF) and glial-derived neurotrophic factor (GDNF). Several modifications of this protocol have been developed to manipulate the percentage of excitatory and inhibitory cell populations and to mimic dorsal vs. ventral signals [12,13]. Such cell cultures contain a mixture of the two types of neurons, which can easily be characterized using standard electrophysiological techniques. Single cell RNA-sequencing (scRNA-seq) has been used to further differentiate these cells into various subtypes [14].

To study PD, differentiation protocols for the midbrain have been developed, specifically for A9-type dopaminergic (DA) neurons, which are the first damaged. hiPSC-derived A9-type DA neurons (abbreviated hiPSC-DA neurons) can be identified by the co-expression of key transcription factors such as LMX1A/FOXA2/NURR1, expression of inwardly rectifying potassium channels (GIRK2), and the capacity to produce pace-maker activity mediated by Cav1.3 calcium channels. Current protocols take advantage of critical transcription factors (e.g., OTX2, LMX1a, FOXa2, LMX1b, MSX1, EN1, NGN2, NURR1, and PITX3) and signaling molecules (e.g., SHH, WNT, and FGF 8) that govern mammalian midbrain development [15,16,17,18]. Most of these methods produce mixed cultures, and the current gold standard for producing midbrain DA neurons from hiPSCs is the floor-plate based method [19]. This robust protocol generates cultures containing over 80% of tyrosine hydroxylase (TH)+ neurons, many of which also exhibit the characteristics of the A9 phenotype described above [19,20,21]. Several improvements to this protocol have been recently described, including the use of specific substrates that enhance the differentiation and function of the neurons produced [22] and transcription factors such as myocyte enhancer factor 2C (MEF2C) to drive A9 differentiation [23].

To study diseases such as PD, Lewy body dementia (LBD), or HD, a protocol for GABAergic medium-spiny neurons (MSN) of the striatum, which synapse with DA neurons projecting from the substantia nigra, was developed [24,25,26,27]. These striatal neurons express DARPP-32 (dopamine and cyclic AMP-regulated phosphoprotein, 32 kDa), and the addition of the Hedgehog agonist purmorphamine and activin A increases the development of these neurons. To date, neurons so generated have been mainly used to study Huntington’s disease [24,25,26,27].

In addition to differentiation protocols for generating neurons, protocols have been developed for differentiating astrocytes using patterning that mimics embryonic development. This entails the addition of agents such as ciliary neurotrophic factor (CNTF), nuclear factor IA (NFIA), and leukemia inhibitory factor (LIF) [28,29,30,31], or the overexpression of various transcription factors, including SOX9 [32]. Many of these protocols require a few consecutive passages to eliminate neuronal cells and achieve a mature astrocytic state.

Microglia, the resident immune cells of the brain, are involved in pathological mechanisms of disease [33]. Neuroinflammation linked to activated microglia has been described in AD [34,35,36,37], PD [38,39,40,41], ALS [42,43], and HD [44]. Since brain microglia originate from the yolk sac rather than the bone marrow similar to other monocytoid cells, to study the involvement of microglia in these diseases, recently, protocols have been developed to recapitulate the differentiation from yolk sac precursors. The aim here is to mimic mature brain-resident microglia using specific signaling molecules, such as bone morphogenic protein 4 (BMP4, a member of the TNF-β superfamily), interleukin (IL)-3 and IL-6, and subsequently granulocyte macrophage colony stimulating factor (GM-CSF) or macrophage colony stimulating factor (M-CSF) and IL-34 [45,46,47,48,49]. Additionally, combinations of cell types can be generated to try to recapitulate some of their interactions in disease pathogenesis.

### 2.2. Three-Dimentional (3D) Models

To better model the brain and understand cell–cell interactions, several procedures have been developed to differentiate neural precursors into a 3D structure in a culture environment. The first 3D systems maintained surface attachment and incorporated several cell types [50,51]. For example, Park et al. developed a 3D tri-culture model system that includes neurons, astrocytes, and microglia in a 3D microfluidic platform [50]. Using this system to study AD, they showed microglial recruitment, neurotoxic activity, such as axonal fragmentation, and nitric oxide (NO) release in AD (vs. wild-type (WT)) cultures that resulted in damage to neurons and astrocytes. Since this system contains several cell types organized in 3D space, it resembles the human brain more closely than 2D mixed cultures and thus may provide a better system to model the disease and develop new therapies.

A more advanced 3D culture system would avoid substrate attachment and expand the cell population as floating 3D organoids [52,53]. Thus, 3D cerebral organoids have recently been used to better understand the complex cellular interactions in a physical structure that resembles the brain more than a co-culture system. Using hiPSCs, many cell types of the brain, including neural progenitor cells, neurons of various subtypes, oligodendrocyte lineage cells, astrocytes, and choroid plexus cells can be generated within cortical organoids, and brain microglia can be incorporated as well [53,54,55,56]. Similar to these cortical organoids, approaches now have been developed to make midbrain 3D organoids [57]. This system is well suited to study PD and LBD; as such, synucleinopathies often begin with neuronal damage in the midbrain. Such specialized culture systems allow the study of brain development and pathology in a context that more closely resembles in vivo brain physiology in humans.

Another progression in the development of 3D organoid culture systems is termed assembloids. Here, several different organoids reflecting different brain regions or tissues are cultured together and allowed to form connections. For example, this allows the study of neuron–muscle interactions [58] or cortex–striatal interactions in an in vitro system [59]. In another example, to better understand different cellular contributions to damage observed during progressive neurodegeneration, there are efforts to include endothelial cells and blood vessels in organoids to model the blood–brain barrier (BBB). The BBB is formed by vascular endothelial cells, pericytes, and astrocyte end-feet, and it enables the separation of systemic blood from cerebrospinal fluid (CSF) [60]. In vitro systems have been developed to model the BBB using endothelial cells and astrocytes [61,62]. Several groups have also been successful in incorporating hiPSC-derived endothelial cells into organoids to create a vasculature. In a recent report, vascularization was accomplished from the outside surrounding matrix as opposed to direct injection into the center of the organoid to recapitulate early fetal vascular brain development. CD31-positive blood vessels were found inside and in between rosettes within the center of the organoid [63]. Another group reported the generation of vessel-like structures with mature BBB characteristics within cerebral organoids after adding vascular endothelial growth factor (VEGF) to the organoid culture medium. Further treatment with VEGF and Wnt7a was added to promote the formation of the outer lining consisting of pericyte-like cells that surrounded the vascular tubes [64].

Another advancement of the 3D culture system is the intracerebral transplantation of hiPSC-derived cerebral organoids into mice. In one example, this resulted in the growth of murine blood vessels into the human tissue, with clear benefits for cell survival and maturation compared with organoids kept in vitro [65]. Moreover, transplanted organoids did not undergo the extensive necrosis seen in vitro with prolonged culture. After transplantation, neurons in the innermost regions of the organoid appeared healthy and produced extensive neural processes decorated with synapses, both within the organoid and in regions of the host cortex where organoid-derived axons projected. Finally, the authors showed evidence of maturing neuronal activity.

## 3. Tools for Drug Discovery

**Figure 1:**
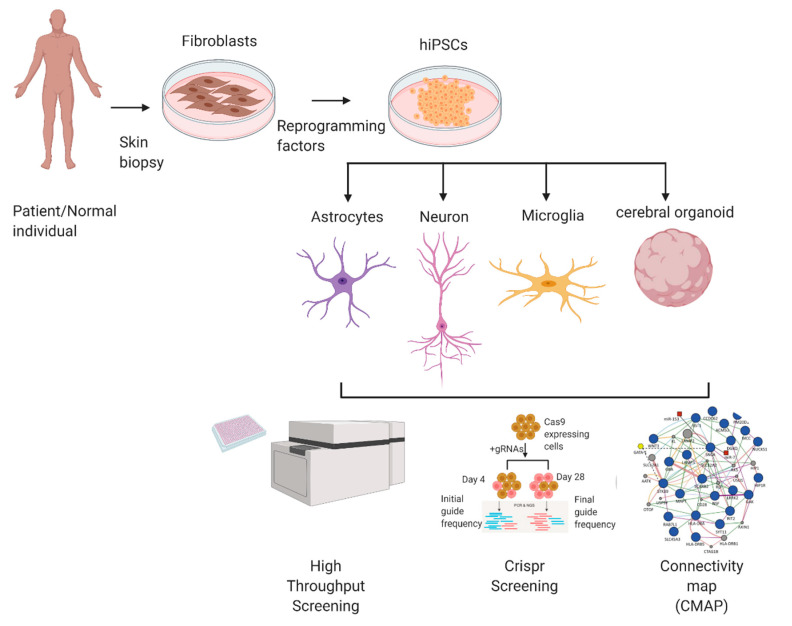
hiPSC-derived brain models in drug discovery. Diagram illustrating the use of hiPSCs to generate various brain cells (neurons, astrocytes, microglia) and 3D cerebral organoids, which can be used to model different neurological disorders. Myelin-forming oligodendrocytes can also be modeled with hiPSC differentiation protocols but are not shown here. These hiPSCs can be used for drug discovery using various methods, e.g., high-throughput screening, CRISPR screening, and Connectivity Map score for transcriptome correction.

hiPSC can be used to model various diseases and can be adapted for a variety of methods to screen for and test potential therapeutic drugs. In this section, we will describe some of the methods to utilize hiPSCs in drug discovery strategies (Figure 1).

### 3.1. High-Throughput Screening

High-throughput screening (HTS) methods have been developed to evaluate large libraries of candidate drugs in a cost and time-efficient method. However, due to a high attrition rate, very few viable drug candidates usually arise from even millions of screened compounds [66]. HTS can investigate hundreds of thousands of compounds per day or more. The screening of compounds that have been culled to yield more likely candidate ‘hits’ could save cost and time. In this regard, recent advances in artificial intelligence (AI) and neural network learning as well as in silico library screening with molecular docking hold the promise for increased screening efficiency. Several readouts may be used for HTS, including fluorescence labeling methods for specific molecular targets, reporter-gene luminescent assays for transcriptional activity, mass spectrometry analysis for proteomic changes, and cell-based phenotypic screens [67]. Another strategy to decrease the time from initial screening to drug development is using drug repurposing. The ReFRAME collection (Repurposing, Focused Rescue, and Accelerated Medchem) is the largest such collection of >13,000 compounds [68]. This collection is composed of FDA- or EMA-approved drugs as well as compounds with demonstrated safety profiles in humans that can be used for other indications.

### 3.2. Connectivity Map

The Connectivity Map (abbreviated “CMAP”) is an emerging approach for drug discovery that can be used in conjunction with hiPSC platforms. It has been used widely for cancer drug discovery and is now being applied to neurological disorders [69,70,71]. CMAP utilizes a collection of transcriptomes obtained from cultured human cells, often from cancers, and it analyzes the changes in their gene expression profiles after treatment with bioactive small molecules to discover relationships between the drugs, gene expression changes, and the phenotypes exhibited by the cells [72]. The CMAP approach uses a gene signature set consisting of differentially expressed genes (DEGs) in a disease vs. control condition as a query, and it compares the DEGs against a large reference catalogue of drug-induced transcriptome changes obtained from a variety of cell lines. By using a pattern-matching algorithm, a similarity metric is obtained between a test gene signature and reference sets, which is transformed to a ‘connectivity score’ ranging from +1 (positive correlation) to −1 (negative correlation) to reflect the connection between the expression profiles [72]. The connectivity score indicates whether the exposure to a particular drug/molecule can accentuate or exacerbate the expression pattern of the gene set of interest.

The use of CMAP for drug discovery in neurodegenerative diseases using transcriptional profiles of hiPSC-based models of the disease has been limited due to the availability of full drug profiles focused on a restricted set of immortalized human cell lines, which do not include hiPSCs. On the other hand, a variety of hiPSC-derived cells such as neural stem cells and differentiated cortical neurons have been used for profiling an extensive drug set as part of the Library of Integrated Network-Based Cellular Signatures (LINCS) project [73]. However, this profiling is based on a set of 1000 landmark genes, which were then used to generate full profiles by optimized linear mapping [73]. Several studies have now started to define CMAP drug candidate expression profiles in additional neurodegenerative disease-relevant cell types such as hiPSC-derived cortical neurons as well as based on the complete transcriptome. In a recent publication, one group established an AD transcriptional profile landscape and assayed a series of candidate drugs from the CMAP database of cancer cell line profiles for their effect on the hiPSC-derived cortical neuron transcriptome [71]. Out of the 153 hiPSC drug profiles, 51 drugs showed a high degree of negative correlation with transcriptional changes observed in AD. By pathway enrichment analysis, these drug candidates were found to upregulate pathways affecting mitochondrial function and downregulate pathways related to immune response. Since pathological features related to mitochondrial function and immune response are common in multiple neurodegenerative disorders, these drug candidates may have wide therapeutic potential [71]. Interestingly, among the 51 drugs encountered using these methods, 18 were already known for their neuroprotective ability, providing confidence in the approach [71]. Although it is encouraging to see progress in the use of computational tools for drug discovery, there is a huge unmet need for generating transcriptomic drug-perturbation databases in hiPSC-derived cell types representing specific brain regions in order to measure drug responses relevant to the cell types affected in various neurodegenerative diseases.

## 4. Disease Modeling Using hiPSCs

### 4.1. Alzheimer’s Disease (AD)

AD is the most common neurodegenerative disorder, and it is characterized by the aggregation of amyloid β (Aβ) and Tau proteins, synaptic loss, neuronal cell death, and resulting memory and cognitive dysfunction [74,75]. Although simulating only limited features of the disease, transgenic mouse models have been the gold standard for studying AD pathophysiology. Despite a significant gain in our knowledge of AD pathophysiological mechanisms from these models, clinical translation remains challenging, resulting in a lack of effective disease-modifying therapy. One issue with these mouse models relates to basic difference in rodent vs. human biology. Reprogramming somatic human cells from AD patients and healthy people (as controls) into hiPSCs, and then differentiating these cells into various brain cell types, has provided an unprecedented opportunity to study disease mechanisms in a human context. Moreover, single genetic mutations causing AD that are amenable CRISPR/Cas9 correction can yield isogenic controls. Additionally, self-organizing 3D cerebral organoids composed of hiPSC-derived cells, which display key features of brain-specific cytoarchitecture and network properties, can be utilized to study complex neural network phenomena in these AD models. Several AD-specific pathophysiological features have been observed using these human in vitro models, which can be used as test therapeutic intervention.

### 4.2. Familial AD (fAD)

fAD presents as an early onset, more aggressive form of AD characterized in most cases by increased Aβ42, Aβ40, and Aβ42/40 ratio. Many cases of fAD result from mutations in the genes encoding amyloid precursor protein (APP) or presenilin 1/2 (PS1/2). Several characteristics of the disease have been observed in hiPSC-derived cerebrocortical neuronal cultures, cerebral organoids, and mixed hiPSC-derived cultures generated from familial AD patients [76,77,78,79]. In addition to neurons, fAD astrocytes and microglia have been shown to display abnormalities, such as reduced capacity to internalize Aβ42 [80,81,82]. In addition to aberrant Aβ production, we have shown that hiPSC-derived cerebrocortical neurons and cerebral organoids manifest hyperexcitability similar to that observed in fAD patients and fAD transgenic mouse models [83,84]. Mechanistically, this hyperexcitability in fAD hiPSC neurons can been attributed to decreased neurite length, synaptic dysfunction, and abnormal ion channel activity in both the 2D and 3D culture systems [50,81,82].

Several reports have also shown that hiPSC-derived neurons bearing the V717F/G mutation in APP manifest elevated Aβ production accompanied by increased soluble (s)APPβ generation [77,85]. This resulted from the mislocalization of APP in early endosomes, accumulation of APP in the endosomes, and increased cleavage of APP by β-secretase enzyme (BACE-1). Enlarged endosomes caused by APP accumulation have been observed in hiPSC neurons with other fAD mutations such as APP duplication (APPdp) [79]. Interestingly, BACE-1 inhibition rescued the defects in endocytosis that were observed across several fAD mutations [85,86]. Additionally, Down syndrome (DS) patient hiPSCs have been used to study AD because DS patients often develop early-onset AD due to triplication of the APP gene as part of trisomy 21 [87]. DS hiPSC-derived neurons display increased Aβ secretion, which could be reversed by deletion of the extra copy of the APP gene [88,89,90,91]. These neurons also exhibited enhanced Tau phosphorylation, which could not be rescued by deletion of the extra copy of the APP gene, suggesting the presence of Aβ-dependent and independent AD phenotypes in DS [91].

Another prominent feature of AD is the presence of hyperphosphorylated Tau (pTau) aggregates, which are known as neurofibrillary tangles. Several hiPSC-derived neuronal models bearing microtubule associated protein tau (MAPT) mutations show tau pathology. However, since tau pathology is observed in later stages of the disease, it is not observed in the usual timeframe (≈2 months) of in vitro neuronal differentiation [92,93]. Although tau pathology, i.e., tangles, was absent from such cultures in vitro, hiPSC neurons from fAD patients did manifest pTau [79,94]. Neurons from hiPSCs bearing an APP duplication (APPdp) exhibited an increase in glycogen synthase kinase (GSK)-3β activity, representing a major Tau kinase, which was rescued by BACE-1 inhibition [79].

Although several fAD related phenotypes were observed in fAD hiPSC-neurons in 2D cultures, they failed to recapitulate robust extracellular amyloid plaques and neurofibrillary tangles; in part, this may have occurred because of low levels of Aβ oligomers, which were decreased further due to regular media changes [95]. However, 3D cerebral organoid cultures as well as 3D matrigel systems have shown increased Aβ aggregation and filamentous hyperphosphorylated tau aggregation, thus providing evidence for their potential use as a model system to understand these disease mechanisms [51,94,95]. In addition to manifesting Aβ and Tau aggregates, cerebral organoids and other 3D systems can recapitulate the hypersynchronous burst-like neural network electrical activity, which is commonly observed in humans during early stages of AD [50,84].

### 4.3. Sporadic AD (sAD)

Unlike hiPSC-derived neurons from fAD patients, most sAD patient-derived neuronal cells fail to manifest a robust increase in Aβ42/40 ratio or pTau levels [96,97]. This could be due to the variability in the genetic background of sAD patients and the contribution of other factors causing sAD. However, hiPSC neurons from two sAD patients have been shown to recapitulate elevated Aβ levels, activated GSK-3β, and pTau [79]. Moreover, others have observed increased aggregation, GSK-3β activity, and pTau in sAD hiPSC-derived neuronal cultures [96]. Additionally, sAD neuronal models consistently manifested endosomal pathology with a decrease in clathrin, which is a protein known to mediate endocytosis [97]. Furthermore, mitochondrial dysfunction and oxidative stress have also been implicated in the pathophysiology of neurons and astrocytes in both fAD and sAD. fAD hiPSC neurons can be used to study the underlying genetically-triggered molecular events leading to the pathophysiology of AD; sAD neurons may reflect a collection of genetic single nucleotide polymorphisms (SNPs) that predispose to AD, allowing the identification of novel AD-associated gene networks [98]. However, it can be argued that sAD might be better modeled by the direct conversion of neurons from fibroblasts (so-called hiN cells), thus avoiding the epigenetic changes that occur with an induced pluripotency stage [79,99].

One of the major genetic risk factors for sAD is the APOE4 allele. Neurons derived from hiPSCs expressing different APOE variants have been used to elucidate sAD-related pathways [100,101,102]. Both astrocytes and neurons express APOE; however, under normal conditions, astrocytes are the main cells expressing APOE in the brain. Neurons express APOE only under stress conditions or trauma [103,104]. Genes associated with lipid metabolism such as phosphate-containing compound metabolic process-associated genes such as CROT, LPGAT1, and PLPP3 were upregulated in APOE4 (APOE4/4) hiPSC-derived astrocytes compared to homozygous APOE3 (APOE3/3) astrocytes [100]. Neurons co-cultured with APOE4/4 astrocytes displayed decreased amounts of neuronal and synaptic markers in addition to increased mortality compared to isogenic cultures. These abnormalities were due to the inability of these astrocytes to maintain neuronal and synaptic homeostasis [101]. APOE4/4 patient hiPSC astrocytes secreted less lipidated APOE lipoprotein particles compared to APOE3/3 astrocytes. Furthermore, isogenic APOE4/4 astrocytes produced and secreted less APOE than APOE3/3 cells [101]. Due to a defect in lysosomes, APOE4/4 astrocytes were less efficient in clearing Aβ42 [100]. Additionally, APOE4/4 hiPSC-derived neurons manifested an increase in Aβ secretion and pTau in 2D cultures and in 3D cerebral organoids [100,102]. APOE4/4 hiPSC neurons also displayed an increase in early endosomes similar to that observed in sAD and fAD hiPSC neurons [100]. These abnormalities could be normalized to a great extent by the genetic correction of APOE4/4 to APOE3/3 [100].

Microglia are the second major contributor of APOE after astrocytes and are often associated with Aβ plaques in the AD brain [105]. Compared to non-APOE4 genotypes, APOE4/4 hiPSC-derived microglia showed an increased inflammatory reaction with an increase in immune response genes, whereas genes involved in cell movement and development were downregulated [100]. APOE4/4 hiPSC microglia also displayed altered morphology compared to APOE3/3 microglia and manifested decreased Aβ42 phagocytosis. Furthermore, when seeded into 3D cerebral organoids, APOE4/4 microglia exhibited decreased ability to sense and engulf Aβ42, which resulted in an increase in extracellular Aβ levels in the organoids [100,106].

These studies and others suggest that hiPSC-derived brain cells can be used to model several aspects of the pathophysiology of AD in a human context. They may also be able to provide novel insights into disease-related mechanisms and serve as a screen for drug discovery, as described further in the next series of sections.

### 4.4. AD hiPSC-Based Models for Drug Discovery

One of the potential applications of hiPSC-based models of AD is to validate/optimize current therapeutic approaches for AD in a human context. To date, one such study employed a fAD hiPSC neuron-based platform bearing a PSEN1(G384A) mutation to test the effect of >1000 compounds on Aβ production. Of these, six lead compounds exhibited dose-dependent Aβ42 reduction; a combination of bromocriptine, cromolyn, and topiramate showed the most potent anti-Aβ effect, which was further validated in other fAD and sAD hiPSC neuronal models [107]. With a similar approach, various antibodies against monomeric and/or oligomeric Aβ species that are currently in human clinical trials based on their efficacy in AD mouse models could be tested for hiPSC-based models of AD. One such study generated a medium-throughput platform to evaluate the effects of Aβ-specific blocking antibodies on the neurite outgrowth of hiPSC neurons exposed to soluble Aβ-enriched AD brain extracts. This study reported that the addition of three Aβ-specific blocking murine antibodies (1C22, 3D6, or 266) decreased Aβ-mediated toxicity and were in fact more effective than the murine-derived immunotherapeutics, bapineuzumab and solanezumab. However, their effectiveness in a human context in comparison to humanized counterparts of the above-mentioned antibodies is yet to be studied [108]. Interestingly, our group recently reported that hiPSC-derived microglia react to the antibody/misfolded protein complexes composed of Aβ or α-synuclein (αSyn, which is found predominantly in PD and LBD but also in AD) with an increased inflammatory signature compared to mouse microglia [49]. This provides evidence that therapies effective in mouse models of AD may not reflect the potential inflammatory side effects found in a human context. While the Biogen anti-amyloid antibody therapy, aducanumab, was recently approved by the FDA for the treatment of AD, this was a contentious review process with mixed data being generated in human trials [109]. Based upon our recent work [49], we speculate that combating the neuroinflammatory response that is engendered by these antibodies when bound to their cognate protein (Aβ) might improve the overall efficacy of AD treatment by them.

In addition to studying toxic Aβ species, 3D culture models such as 3D cerebral organoids have been used to test if specific antibodies can block tau pathology [50,95,110,111,112]. Determination of the efficacy and optimal dosage of small molecule drug candidates can also be performed using hiPSC-based AD models. As outcome measures, such studies can monitor changes in Aβ and/or pTau levels, misfolded-protein associated toxicity, or pathological neuronal hyperactivity, as this parameter has been shown to contribute to synaptic damage and neuronal cell death [84,95]. For example, nonsteroidal anti-inflammatory drug (NSAID)-based γ-secretase modulation was shown not to be as effective in hiPSC-derived neuron cultures as in rodent neurons, possibly reflecting its failure in human clinical trials for AD [95,113]. In contrast, other drugs aimed at decreasing aberrant neural network activity in AD, such as NitroSynapsin, showed a positive outcome in hiPSC-derived neuronal cultures and cerebral organoids [84,114]. NitroSynapsin abrogated the hyperexcitability observed in hiPSC-derived neurons bearing the PS1 ΔE9 mutation, PS1M146V mutation, or APPswe mutation [84]. Among other drugs that showed promise in hiPSC-based AD models is the small molecule, PH002, which changes the conformation of APOE4 to resemble the conformation of APOE3, leading to the restoration of physiological Aβ40/Aβ42 ratios and decreased pTau levels [102].

Recently, by performing multi-omics analyses, using methods such as whole-genome sequencing (WGS), RNA-seq, and single-nucleus RNA-seq (snRNA-seq) analyzing postmortem human brain samples from late onset AD (LOAD), ATP6V1A was predicted to be a key regulator of a neuron-specific subnetwork most affected by LOAD [115]. In contrast, by using hiPSC-derived cerebral organoids, we can interrogate early stages of the disease for molecular modules that may represent novel therapeutic targets, critically, at an earlier stage of the disease when effective treatment can still be initiated.

### 4.5. Parkinson’s Disease (PD)

PD is the second most prevalent neurodegenerative disorder; it can be either familial or sporadic in nature. Pathophysiologically, PD is characterized initially by the loss of DA neurons in the substantia nigra pars compacta of the midbrain, leading to compromised motor functions including tremor, rigidity, and paucity of movement. The most common nonmotor symptom of PD is dementia, with a prevalence of about 80% [116]. Moreover, a subset of PD patients also exhibit additional cognitive impairments, including deficits in language, executive function, and psychiatric symptoms, such as hallucinations and delusions, particularly in patients with Lewy body dementia (LBD) [117]. While advanced age, oxidative/nitrsoative stress, and other environmental factors and toxins have been implicated in sporadic PD, several causative genes have been associated with familial PD [118]. Before the advent of hiPSCs, the commonly used animal and cellular models for PD were either developed using toxins such as rotenone, MPTP, 6-OHDA, or genetic techniques [119,120,121,122,123]. Although toxin-based models can recapitulate DA neuronal degeneration and motor deficits, their non-specificity and side effects are major drawbacks. Synaptic loss may precede neuronal cell death but is not always examined in these model systems. On the other hand, genetic models, including mutation or knock out of the genes encoding αSyn, parkin (a ubiquitin E3 ligase), PTEN-induced kinase 1 (PINK1, a mitochondrial serine/threonine-protein kinase), DJ-1 (a protein deglycase), leucine-rich repeat kinase 2 (LRRK2), and glucocerebrosidase (GBA), have been generated to gain insight into PD pathophysiology [124,125,126,127]. However, the species-specific differences between neurodegenerative phenotypes observed in animal models vs. humans make the results less conclusive and clinically translatable. In addition, many animal models do not recapitulate all of the features of PD such as Lewy bodies and nonmotor symptoms. This has led to a lack of disease-modifying treatments aimed at delaying demise or even rescuing DA neurons. Some treatments such as levodopa and dopamine agonists offer symptomatic relief, and deep brain stimulation may result in prolonged improvements in motor function and decreased dosage of dopamine agonist drugs for PD patients. While dopamine agonists help with bradykinesia for a number of years, their effectiveness eventually wanes, making cell replacement therapy important for future intervention [128,129]. The monoamine oxidase (MAO)B inhibitor rasagiline has been reported to slow disease progression [130], but this effect remains contentious. In any event, these treatments are far from ideal and there is still room for improvement to develop more effective therapies against PD. hiPSC technologies have enabled the generation of DA neurons from individuals who suffer from familial or sporadic PD and allowed the study of interactions between genetic and exogenous factors involved in PD pathogenesis, the development of new drug therapies, and early clinical trials for cell replacement therapy [131]. A combination of cell replacement therapy with other approved drugs could prevent or delay further recurrence of the neuronal damage.

### 4.6. Familial PD (fPD) Modeling

Several studies have shown that hiPSC-DA neurons bearing various PD-causing genetic mutations manifest several pathophysiological features of the disease, as observed in patients. A critical feature of PD is the appearance of Lewy bodies, which are comprised of aggregated and/or misfolded αSyn as well as additional misfolded proteins. Several mutations in SNCA, the gene encoding αSyn, including A53T and E46K, as well as duplications and triplications have been described in familial forms of PD.

Similarly, SNCA point mutations and triplications have been found to cause accumulation of αSyn protein in hiPSC-DA neurons [20,132]. hiPSC-derived DA neurons bearing triplicate SNCA produced twice the normal levels of αSyn, overexpressed oxidative stress markers, and manifested lysosomal dysfunction [133,134]. Furthermore, the presence of high levels of αSyn in these neurons was associated with decreased axonal density and synaptic degeneration [135]. Importantly, the observed pathology could be reversed by knocking out endogenous αSyn in hiPSC-DA neurons bearing the triplicated SNCA gene [136]. Similar to the effect of triplicated SNCA, hiPSC-DA neurons with A53T or E46K point mutations in the SNCA gene exhibited αSyn aggregation resembling Lewy body-like pathology, dysfunctional mitochondrial axonal transport, and abnormally increased reactive oxygen and nitrogen species (ROS/RNS) [20,133,134]. The resulting nitrosative stress was found to cause aberrant protein S-nitrosylation of transcription factor MEF2C, leading to peroxisome proliferator-activated receptor γ coactivator (PGC)-1α-mediated mitochondrial dysfunction and apoptotic cell death in hiPSC-DA neurons [20]. Several models for αSyn transmission have been described, using an external source of αSyn (in the absence of mutation) [137,138]. Recently, using hiPSC-derived neurons, disparate strains of αSyn induced different degrees of neuronal damage and propagation. Fibrils and ribbons were transported between neurons and induced mitochondrial damage [139]. Moreover, αSyn oligomers disrupted anterograde axonal transport of mitochondria as a result of subcellular changes in transport-regulating proteins and energy deficits, with consequent synaptic degeneration [140]. Finally, we have recently shown that αSyn oligomers induce extrasynaptic NMDAR activity via both direct effects on the receptor and astrocytic glutamate release, contributing to synaptic loss in hiPSC-derived neurons [141].

Another gene implicated in fPD is LRRK2, with mutations associated with both familial and sporadic forms. More than 50 different missense and nonsense mutations in LRRK2 have been reported to date [142], most of which manifest gain of function attributes. Of those, the G2019S mutation has been extensively studied [143,144]. Mitochondrial dysfunction, autophagy defects, and DA neuronal degeneration were observed in hiPSC-DA neurons bearing the LRRK2 mutation G2019 [145,146,147,148]. Other LRRK2 mutations such as G2385R, R1628P, N551K, and S1647T show similar mitochondrial defects, disrupted calcium homeostasis, abnormal synaptic vesicle trafficking, and other PD-related phenotypes [149,150,151]. Three-dimensional (3D) midbrain organoids bearing the LRRK2-G2019S mutation, when compared to isogenic, gene-corrected controls, recapitulated several PD-related pathological features [147]. These mutant organoids exhibited an increase in mitophagy, accumulation of phosphorylated αSyn in endosomes, and neuronal damage [147].

PINK1 and Parkin mutations in hiPSC-DA neurons cause neurite degeneration, DNA damage, increased mitochondrial ROS, and mitochondrial enlargement with inclusions [152,153,154]. Interestingly, S-nitrosylation of PINK1 in hiPSC-DA neurons produced deficits in mitophagy similar to those observed with familial PINK1 mutations [155]. Additionally, fPD patient-derived hiPSC-DA neurons bearing SNCA, LRRK2, or Parkin mutations manifested disrupted synaptogenesis, synaptic transmission, and neurotransmitter release [133,149,150].

In addition to neurons, other cell types derived from fPD hiPSCs also affected PD-related pathology. For example, hiPSC-derived astrocytes bearing the LRRK2(G2019S) mutation manifested dysregulated autophagy. Activated hiPSC-derived microglia from the same precursors caused a decrease in neurite length in hiPSC-DA neurons via aberrant IFN-γ signaling [156]. Parkinson’s patient-derived hiPSC neural cells with a mutation in the OPA1 gene, which has been associated with fPD, showed dysfunction in mitochondrial dynamics, increased oxidative stress, and inflammation with induction of neuronal necroptosis [157]. These studies suggest that many fPD-associated mutations in hiPSC cell-based models can recapitulate key aspects of PD pathophysiology.

### 4.7. Sporadic PD (sPD) Modeling

In comparison to hiPSC-DA neurons from healthy individuals, sPD patient-derived hiPSC-DA neurons displayed increased expression of cleaved caspase-3, shortened neurite length, increased aggregation of αSyn, and defective autophagosome clearance after long-term culture [158,159]. Interestingly, genome-wide DNA methylation studies on sPD hiPSC-DA neurons showed alterations in epigenetic signatures, leading to changes in the expression pattern of several genes [160,161]. These abnormalities in the epigenome were very similar to those found in LRRK2-associated PD patient-derived hiPSC-DA neurons [160]. Interestingly, these abnormalities did not appear in parental skin cells, undifferentiated hiPSCs, or other hiPSC-derived neural cells, suggesting that they are specific to the differentiation process of dopaminergic cells. Hypermethylation was prominent in gene regulatory regions, such as enhancers, and it was correlated with RNA and/or protein downregulation of a network of transcription factors relevant to PD (FOXA1, NR3C1, HNF4A, and FOSL2) [160]. Another extensive transcriptomic and epigenomic study on sPD hiPSC-DA neurons found alterations in gene expression in CREB and the PGC-1α mitochondrial pathway, which are known to be involved in PD pathogenesis [162]. Furthermore, the authors suggested that sPD hiPSC-DA neurons show differential regulation of miRNA and piRNA molecules, which was also observed in human PD brain postmortem tissue samples [162]. sPD hiPSC-DA neurons also exhibited disrupted synaptic transmission and delayed firing synchronicity, which was accompanied by decreased spontaneous activity [158]. Therefore, hiPSC-derived neurons from sPD patients can potentially provide a mechanistic understanding of PD pathophysiology and help identify novel pathways that can be targeted for therapeutic intervention.

If consistent phenotypes are observed in neurons and glial cells derived from hiPSCs with single fPD-associated gene mutations, when compared to isogenic controls on multiple genetic backgrounds, the results can be quite reliable and robust. However, no single genetic mutation manifests 100% penetrance in PD [123]. Instead, the pathophysiology of both fPD and sPD is most likely dependent on multiple genetic risk factors that act in conjunction with aging and environmental factors.

### 4.8. PD hiPSC-Based Models for Drug Discovery

PD hiPSC-based models have been used for both drug discovery and cell-based therapies to treat PD. For example, valinomycin- or concanamycin A-induced cytotoxicity in PD patient hiPSC-derived neurons was rescued by various drugs, including the mitochondrial oxidative phosphorylation-enhancer coenzyme Q10, LRRK2 kinase inhibitor GW5074, and immunosuppressant and mTOR pathway inhibitor rapamycin [154]. Furthermore, rapamycin and GW5074 selectively decreased the production of ROS in hiPSC-derived neurons bearing a PINK1 mutation vs. wild-type controls, thus highlighting the differential susceptibility of diseased vs. healthy neurons to pharmaceutical agents [154]. A small molecule, isoxazole, identified by HTS of small molecule libraries, was found to abate the mitochondrial dysfunction and apoptotic cell death at least in part by increasing the activity of the MEF2C-PGC-1α transcriptional network in hiPSC-DA neurons bearing an A53T αSyn mutation [20].

At least two other studies have developed phenotypic screens of hiPSC-DA neurons from fPD patients bearing parkin/PINK1 mutations. One of the studies showed that hiPSC-DA neurons derived from patients with a mutation in PARK2 (which encodes the E3 ligase parkin) displayed increased mitochondrial stress after exposure to rotenone, as identified by increased cleaved/activated caspase-3 (casp3) levels [163]. From the phenotypic screening of 1165 compounds from an FDA-approved drug library, 88 compounds were selected based on their ability to decrease cleaved casp3 levels [163]. Among these compounds, a voltage-gated T-type calcium channel antagonist, benidipine, prevented rotenone-induced apoptosis and showed other neuroprotective effects [163]. This study described a novel pathological mechanism in PD that could be potentially exploited for treatment [163]. The second study used a semi-automatic, high-throughput imaging-based quantitative assay for detecting mitochondrial clearance and cell viability of hiPSC-DA neurons. Among 320 pharmacologically active inhibitor compounds screened, the study identified four hits, tranylcypromine, bromocriptine, MRS1220, and flunarizine, that promoted the lysosomal degradation of mitochondria and hence improved mitochondrial clearance [164]. Similar to the above phenotypic screenings, a third study performed the screening of 273 small molecule kinase inhibitors on A53T-αSyn hiPSC-DA neurons. They identified BX795, a multikinase inhibitor, as a drug candidate that rescued PD pathology by robustly increasing TH expression [165].

Cell-replacement therapy for the treatment of PD has shown variable levels of success in patient in the past using various cell types, including fetal cells [166]. Transplantation strategies in PD are intended to replace the loss of DA neurons in the nigrostriatal system. Recently, human embryonic stem cell (hESC), hiPSC, and direct lineage reprogramming-based approaches have been used for cell replacement therapy in preclinical studies for PD treatment. Indeed, these studies have reported successful brain transplantation of hiPSC-DA progenitor cells in both rodent and primate models of PD [167]. Interestingly, these cells survived and extended dense neurites into the striatum, where they were implanted, to form connections without causing substantial side effects or tumor formation for two years, leading to a significant improvement in spontaneous movement in these animals. In another study, the transplantation of major histocompatibility complex (MHC)-matched monkey iPSCs in the PD monkey brain resulted in decreased neuroinflammation compared to non-MHC matched cells [168]. These preclinical studies suggest that hiPSCs may be used to develop cell replacement therapies for PD. Such studies in humans have recently begun with hESC and hiPSC-DA progenitors [131].

### 4.9. Amyotrophic Lateral Sclerosis (ALS)

Amyotrophic lateral sclerosis (ALS) is characterized by the progressive degeneration of upper and lower motor neurons (MN). While ≈90% of ALS patients are sporadic cases (sALS), some 5–10% are familial ALS (fALS), which in most cases is caused by the genetic inheritance of autosomal dominant mutations. The most common mutations causing ALS occur in genes such as superoxide dismutase 1 (SOD1), transactive response DNA-binding protein 43 (TARDBP, which encodes the protein TDP-43), hexanucleotide repeat expansion in the chromosome 9 open reading frame 72 (C9ORF72), and fused in sarcoma (FUS). Although these genetic factors causing ALS are known, there are ≈30% of fALS cases where the genetic etiology is not yet known [169]. Additionally, the mechanism(s) underlying pathophysiology in sALS is also poorly understood. The slow progress in understanding ALS disease mechanisms has occurred in part because of a lack of robust ALS models. Transgenic murine models of fALS have been extensively used to study the pathological mechanisms underlying ALS in an attempt to develop therapies. However, as with other neurodegenerative disease modeling, drugs developed from the animal models have shown little or no efficacy in human trials, reflecting, in part, crucial interspecies differences. Additionally, transgenic animal models cannot be used to investigate sALS cases, which constitute the majority of ALS patients. Postmortem human tissue samples have enabled studies of ALS pathology in a human context; however, insufficient postmortem tissues coupled with the fact that many neurons have died by the time such samples become available have further hindered progress. Moreover, postmortem tissues are primarily from late-stage ALS patients and therefore do not provide the opportunity to study the initial stages of the disease [170,171].

### 4.10. Familial ALS (fALS) Modeling with hiPSCs

One of the most common types of fALS arises from mutations in the SOD1 gene, which has been extensively studied in transgenic mice. fALS patient hiPSC-derived MN (hiPSC-MN) carrying SOD1 mutations have revealed a proapoptotic phenotype and increased degeneration. Additionally, the transcriptomic analysis of hiPSC-MN showed significant transcriptional changes associated with increased oxidative stress, mitochondrial dysfunction, cytoskeletal disruption, ER stress, and activation of the unfolded protein response [172,173]. Another study demonstrated that patient hiPSC-MN carrying the SOD1 mutation (A4V) were consistently hyperactive compared to isogenic, gene-corrected control neurons [174]. In contrast to this observation, hiPSC-MN bearing another SOD1 mutation (D90A) manifested hypoexcitability in combination with lower Na^+^/K^+^ current ratios. Although it is not clear whether either hyper- or hypoexcitability can play a crucial role in ALS pathology, major electrophysiological changes were shown to occur in hiPSC-MN, which may represent potential therapeutic targets [174]. Interestingly, hiPSC-derived MNs with a SOD1 (G93A) missense mutation, generated by CRISPR/Cas9, developed axonal pathologies, such as axonal swellings with a shorter axon length and fewer branch points, in addition to abnormalities in presynaptic and postsynaptic size and density. The SOD1 mutation also led to a decrease in the frequency of action potentials and network bursting; however, burst duration increased [175]. Additionally, SOD1 mutant hiPSC-derived astrocytes have been shown to exhibit the upregulation of several proinflammatory genes in parallel to the downregulation of many genes associated with homeostatic functions [176]. SOD1 mutant hiPSC-astrocytes also expressed decreased levels of the inward rectifying potassium channel, Kir4.1, which is implicated in regulating the biophysical properties of fast-firing MN through an astrocyte–neuron interaction. This finding may in part account for dysfunction of fast-firing MN in ALS [177].

Another set of studies examined mutant TARDBP hiPSC-MN, which showed significant ALS-related pathological features, including decreased neurite length, increased oxidative stress, decreased MN viability, and increased soluble as well as insoluble TDP-43 protein compared to healthy control cells [178]. At early stages of TDP-43 mutant hiPSC-MN development in vitro, electrophysiological studies revealed hyperexcitability followed by a progressive decrease in synaptic activity and action potential frequency [179]. These results suggest that the hyperexcitability phenotype is common between hiPSC MN with some SOD1 mutations and TDP-43 mutations. Additionally, hiPSC-astrocytes bearing the TDP-43(M337V) mutation showed cytoplasmic mislocalization of soluble TDP-43 [180]. However, in contrast to mutant hiPSC-MN, the astrocytes did not display any accumulation of insoluble TDP-43, and the survival of co-cultured wild-type hiPSC-MN was not affected.

With rare TDP-43 mutations, such as TDP-43(A90V), which is a risk factor for both ALS and frontotemporal dementia (FTD), hiPSC-MN showed mislocalized cytoplasmic TDP-43. In addition, these hiPSC-MN manifested a decrease in miR-9 expression similar to hiPSC-MN bearing the TDP-43(M337V) mutation, suggesting that miRNA dysregulation may occur as a factor in the ALS/FTD spectrum of disorders [181].

Similar to SOD1 and TDP-43, FUS mutant hiPSC-MN bearing a frameshift mutation at residue 511 (M511FS) or a H517Q point mutation were shown to be hyperexcitable [174]. Conversely, hiPSC-MN bearing a point mutation (R521L or R521C) or a frame shift mutation (R495QfsX527) in the FUS gene were hypoexcitable [182]. These differences in electrophysiological phenotype were also observed in other ALS patient hiPSC-MN. Among other pathophysiological phenotypes, cytoplasmic mislocalization and the formation of FUS aggregates was observed in hiPSC-MN differentiated from a patient carrying the FUS(P525L) mutation [183]. Similarly, other FUS mutant hiPSC-MN showed aberrant cytoplasmic FUS localization and stress granule formation [184]. Furthermore, some defects in hiPSC-MN bearing FUS mutations could be ameliorated pharmacologically by histone deacetylase 6 (HDAC6) inhibitors or genetic silencing, suggesting a potential therapeutic strategy for ALS [185].

Another genetic risk factor for ALS is the expansion of G4C2 repeats in the gene C9ORF72. In this case, hiPSC-based models have proved useful in studying possible pathophysiological pathways. Several studies have shown that C9ORF72 patient-derived hiPSC-MN can recapitulate major pathological aspects of the disease. For example, C9ORF72 mutant hiPSC-MN showed that the expanded RNA repeats sequestered RNA binding proteins, including hnRNPA1, ADARB2, and Pur-α [186,187]. Moreover, C9ORF72 mutant hiPSC-MN manifested dipeptide repeat proteins (DPRs) produced by repeat-associated non-ATG translation; they also exhibited cell-to-cell spreading of DPRs in a co-culture system with control hiPSC-MN [188,189]. Furthermore, C9ORF72 mutant hiPSC-MN displayed disrupted calcium homeostasis, decreased mitochondrial membrane potential, and elevated ER stress [190,191]. Additionally, C9ORF72 hiPSC-MN showed an age-dependent increase in yH2AX, a DNA damage marker [192]. Pharmacological or genetic reduction of oxidative stress partially reduced DNA damage in these motor neurons, suggesting that oxidative stress is an important player in the pathology [192].

Several studies of C9ORF72 mutant hiPSC-MN neurons showed alterations in basal autophagy and p62, an autophagic marker, similar to observations in C9ORF72 patients [191,193,194,195,196]. In line with other mutations underlying ALS, there are reports from electrophysiological studies of C9ORF72 hiPSC-MN showing either increased or decreased network excitability. However, all mutations caused a defect in the electrical properties of these neurons, suggesting that either increased or decreased activity may be pathological [187]. In addition to the known pathological features of ALS, such as disrupted autophagy and endosomal trafficking, studies of ALS patient-derived hiPSC-MS have also revealed novel mechanisms such as impaired nucleocytoplasmic transport [197,198].

In addition to neurons, hiPSC-derived astrocytes were shown to be significantly affected by ALS-related C9ORF72 mutations. For example, C9ORF72 mutant hiPSC-astrocytes exerted toxic effects on co-cultured normal motor neurons [199]. Similarly, media conditioned by C9ORF72 hiPSC astrocytes caused increased oxidative stress in hESC-MN [200]. Moreover, C9ORF72 hiPSC astrocytes displayed an increase in oxidative stress and a decrease in antioxidant protein secretion themselves [200]. Reminiscent of astrocytes, patient-derived C9ORF72 hiPSC-oligodendrocytes or their conditioned medium induced death in normal hiPSC-MN in culture [201]. Finally, recent studies showed that hiPSC-microglia manifest the C9ORF72-associated phenotype of increased DPR translation and reduced C9ORF72 protein levels, despite the fact that their transcriptional profile did not differ from control hiPSC-microglia. Interestingly, C9ORF72 hiPSC-microglia also exhibited intrinsic dysfunction of phagocytic and endosomal–lysosomal pathway activity [202].

### 4.11. Sporadic ALS (sALS)

Sporadic forms of ALS account for the majority of cases. Historically, there has been a lack of good model systems to study sporadic disease because the etiology remains unknown. With the advent of sALS patient hiPSC models, it was found that some pathological and electrophysiological phenotypes of the disease were preserved. For example, one study generated hiPSCs from 16 sALS patients and found spontaneous aggregates of hyperphosphorylated TDP-43 in hiPSC-MN [203]. Another large gene profiling study of hiPSC-MN derived from sALS patients showed the dysregulation of genes associated with mitochondrial function [204]. Additionally, a study on SOD1 mutant patient-derived hiPSC-MN showed an increase in apoptotic neuronal cell death involving the Src/c-Abl pathway and disrupted autophagy; boosting autophagy decreased mutant SOD1 levels, corrected mitochondrial gene expression, and increased the survival of both fALS and sALS-derived hiPSC-MN [205]. A later study done on a much larger number of sALS patient-derived hiPSC-MN lines revealed the cytosolic aggregation of TDP-43, decreased neurite outgrowth, dysfunctional mitochondria, and enhanced ROS production, confirming and expanding the previous studies [206]. Interestingly, the intensity of the phenotype varied according to the clinical severity of ALS in those patients [206].

### 4.12. ALS hiPSC-Based Models for Drug Discovery

The aforementioned studies showed that both fALS and sALS hiPSC-MN exhibit certain features of the disease, affording an opportunity to use these models for drug discovery [207]. For example, retigabine, a clinically approved anticonvulsant that activates subthreshold KCNQ (Kv7) currents, led to a decrease in hyperexcitability and an increase in the viability of ALS patient-derived hiPSC-MN. This study laid the foundation for a clinical trial of retigabine for ALS [174,207]. In an early human trial analyzing the acute effects of the drug in 18 ALS patients, retigabine caused a significant decrease in various parameters of hyperexcitability of motor nerves, thus showing promise as a potential therapeutic [208]. Other drugs screened using ALS hiPSC-MN include anacardic acid, a histone acetyltransferase inhibitor, which decreased TDP-43 aggregation, neuronal susceptibility to toxins, and metabolism-related dysfunction, while increasing neurite outgrowth [178,209]. In C9ORF72 mutant hiPSC-MN, antisense oligonucleotides (ASOs) against C9ORF72 also decreased ALS-associated pathology, which has led to clinical trials [186,187].

As another approach, HTS for drugs has also been used on ALS hiPSC-MN in attempt to develop ALS therapeutics. For example, a drug screening campaign using hiPSC-MN from one sALS patient tested 1757 bioactive compounds, efficacy of the compounds being determined by their potential to decrease the percentage of neurons containing TDP-43 aggregates [203]. Four compounds, including cyclin-dependent kinase inhibitors and the cardiac glycosides/Na^+^/K^+^-ATPase inhibitors digoxin, lanatoside C, and proscillaridin A, were found to be effective in this assay in a dose-dependent manner [203]. Another HTS of 1416 compounds, including existing commercially available drugs or those undergoing clinical testing, led to 27 ‘hits’ that exhibited neuroprotection in ALS patient-derived hiPSC-MN. Among them, bosutinib was found to be most efficacious. Bosutinib decreased the phosphorylation of Src/c-Abl, which in turn led to increased autophagy, thus decreasing the load of misfolded proteins while increasing the expression of tricarboxylic acid (TCA) cycle and respiratory electron transport chain (ECA)-associated genes [205]. More recently, among 1232 FDA-approved drugs screened on ALS hiPSC-MN, ropinirole, a dopamine D2 receptor (D2R) agonist, was found to rescue ALS-related pathologies such as FUS/TDP-43 mislocalization, stress granule formation, neurite retraction, and MN degeneration. Another recent study used a chemogenomic library of 2899 compounds, 67 of which reduced the hyperexcitability of ALS motor neurons carrying the SOD1(A4V) mutation. The targets include two known ALS excitability modulators, AMPA receptors, Kv7.2/3 ion channels, and D2 dopamine receptors as modulators [210]. These studies provide evidence for the successful use of hiPSCs for screening or existing drugs for advancement to clinical trials for the potential treatment of ALS.

## 5. Limitations of hiPSC-Based Models of Neurodegenerative Disorders

As discussed in previous sections, hiPSCs are now being used extensively for the study of neurodegenerative disease mechanisms. One reason for this is the ability to differentiate various patient-derived or mutated hiPSCs to yield homogenous and reproducible cultures of various types of cells found in the brain that can be used for immunostaining, electrophysiology, and multiomic (e.g., transcriptomic, proteomic, and metabolomic) studies. While methods have improved over the years, and there are protocols that produce homogenous populations, this is still a challenge in the field, particularly when using cells of various origins (e.g., hiPSCs derived from blood, skin fibroblasts, or ESCs), and different genetic background. It is important to use isogenic controls, when possible, as well as multiple control and patient lines, to account for the variability and differences in genetic background. In addition, to increase reproducibility, several clones from each hiPSC source, as well as multiple different differentiations are necessary [211].

However, the major drawbacks of this system are the lack of an in vivo physiological environment and the relatively short-term cultures that are available for standard 2D culture systems. Even more complex cerebral organoids can be cultured for only approximately a year or so. This makes the hiPSC models less valuable in predicting events in human patients suffering from age-related diseases. Additionally, similar to other 2D culture systems, cell–cell interactions in hiPSC-derived cultures are largely limited to side-by-side contact and lack relevant cell–extracellular matrix (ECM) interactions. The morphology and gene expression of the cells are also altered in these cultures and hence do not faithfully represent in vivo characteristics.

Some of these limitations can be circumvented by using 3D cerebral organoid models, which allow more complex interactions between the various types of cells found in the brain, including different subtypes of neurons, astrocytes, oligodendrocytes, microglia, and endothelial cells. Organoids provide a more physiological spatial organization that recapitulates to some extent the higher degree of complexity and structure observed in brain tissues. However, the brain organoid system is prone to the heterogenous distribution of oxygen and nutrients required for cell growth, differentiation, and function, reflecting the lack of a vascular system and hence asymmetric distribution of these critical components that can lead to necrotic central areas of cell death or uneven development [212]. Recently, there has been considerable research intended to improve the reproducibility of cerebral organoid generation to mitigate such issues [213,214]. Additionally, scaffold-based systems can be applied to provide specific chemical and physical cues for better cell growth and function [215,216,217,218,219]. However, for scaffold-based approaches, careful consideration needs to be given to material properties of the scaffold (e.g., pore size, chemical composition, and biodegradability), and the process of retrieving cells for various applications [220,221]. Another way to generate a more “in vivo” system is to transplant hiPSC-derived brain cells/organoids into the brains of immunodeficient mice, which are immunodeficient so that the human cells are not rejected [65,222,223,224]. Although this system can allow the study of human brain cells in an in vivo context and with an ingrowing vascular supply, there will be human–mouse cell interactions that may be different than in humans.

Apart from the limitations of the model systems themselves, another major concern related to hiPSC-derived models of neurodegenerative diseases is the modeling of sporadic forms of these diseases. The heterogeneous nature of sAD, sPD, and sALS and the lack of isogenic controls makes it difficult to pinpoint the causal mechanisms for the underlying pathophysiology. High genetic heterogeneity in sporadic disease contributes to multiple cellular phenotypes and therefore requires the investigation of a large number of hiPSC lines vs. controls to obtain statistical power in deriving conclusions about disease mechanisms not simply related to variations in genetic background, for example [225]. Another important factor that needs to be considered is that most epigenetic modifications in donor fibroblasts, with the exception of genetic imprinting or epigenetic memory, are reset to the embryonic state during the reprogramming of fibroblasts to hiPSCs [226,227]. Therefore, the hiPSC-based models of sporadic cases of neurodegenerative diseases demonstrate primarily genetic information-based pathologies for each sporadic case, without the consideration of possible developmental or environmental contributors. To translate results from these models into therapeutics that are successful in clinical trials, these findings need to be validated in a large number of cell lines, which has now become more feasible because of an expanding number of hiPSC lines and recent advances in high-content screening techniques. Other approaches, where fibroblasts have been directly reprogrammed to produce induced neurons (iNs), circumvent some of the issues by maintaining epigenetic signatures of the donor cell. These cells are being widely used for studying late-onset neurodegenerative and aging related disease mechanisms [228,229,230,231].

Other obvious limitations of the hiPSC-derived models of neurodegenerative diseases are the lack of environmental factors and age-related phenotypes. However, it is possible that the stress of an organoid culture may in fact recapitulate some effects of environment and age [232,233]. The resulting aberrant phenotypes resulting from stress could lead to expedited aging in brain organoids, making them in fact a better system to study neurodegenerative and aging disorders. Despite the current limitations of studying neurodegenerative disorders in hiPSC-derived models, several AD, PD, and ALS patient-like phenotypes have been reported in familial and sporadic hiPSC-derived brain cells, thus demonstrating their value in neurodegenerative disease research.

## Data Availability

Not applicable.

## References

[1] Takahashi K., Tanabe K., Ohnuki M., Narita M., Ichisaka T., Tomoda K., Yamanaka S. (2007). Induction of pluripotent stem cells from adult human fibroblasts by defined factors. Cell.

[2] Fusaki N., Ban H., Nishiyama A., Saeki K., Hasegawa M. (2009). Efficient induction of transgene-free human pluripotent stem cells using a vector based on Sendai virus, an RNA virus that does not integrate into the host genome. Proc. Jpn. Acad. Ser. B Phys. Biol. Sci..

[3] Hou P., Li Y., Zhang X., Liu C., Guan J., Li H., Zhao T., Ye J., Yang W., Liu K. (2013). Pluripotent stem cells induced from mouse somatic cells by small-molecule compounds. Science.

[4] Ban H., Nishishita N., Fusaki N., Tabata T., Saeki K., Shikamura M., Takada N., Inoue M., Hasegawa M., Kawamata S. (2011). Efficient generation of transgene-free human induced pluripotent stem cells (iPSCs) by temperature-sensitive Sendai virus vectors. Proc. Natl. Acad. Sci. USA.

[5] Warren L., Manos P.D., Ahfeldt T., Loh Y.H., Li H., Lau F., Ebina W., Mandal P.K., Smith Z.D., Meissner A. (2010). Highly efficient reprogramming to pluripotency and directed differentiation of human cells with synthetic modified mRNA. Cell Stem Cell.

[6] Lin T., Ambasudhan R., Yuan X., Li W., Hilcove S., Abujarour R., Lin X., Hahm H.S., Hao E., Hayek A. (2009). A chemical platform for improved induction of human iPSCs. Nat. Methods.

[7] Zhu S., Li W., Zhou H., Wei W., Ambasudhan R., Lin T., Kim J., Zhang K., Ding S. (2010). Reprogramming of human primary somatic cells by OCT4 and chemical compounds. Cell Stem Cell.

[8] Okita K., Matsumura Y., Sato Y., Okada A., Morizane A., Okamoto S., Hong H., Nakagawa M., Tanabe K., Tezuka K. (2011). A more efficient method to generate integration-free human iPS cells. Nat. Methods.

[9] Zhou H., Wu S., Joo J.Y., Zhu S., Han D.W., Lin T., Trauger S., Bien G., Yao S., Zhu Y. (2009). Generation of induced pluripotent stem cells using recombinant proteins. Cell Stem Cell.

[10] Muotri A.R., Chu V.T., Marchetto M.C., Deng W., Moran J.V., Gage F.H. (2005). Somatic mosaicism in neuronal precursor cells mediated by L1 retrotransposition. Nature.

[11] Chambers S.M., Fasano C.A., Papapetrou E.P., Tomishima M., Sadelain M., Studer L. (2009). Highly efficient neural conversion of human ES and iPS cells by dual inhibition of SMAD signaling. Nat. Biotechnol..

[12] Li X.J., Zhang X., Johnson M.A., Wang Z.B., Lavaute T., Zhang S.C. (2009). Coordination of sonic hedgehog and Wnt signaling determines ventral and dorsal telencephalic neuron types from human embryonic stem cells. Development.

[13] Pasca S.P., Portmann T., Voineagu I., Yazawa M., Shcheglovitov A., Pasca A.M., Cord B., Palmer T.D., Chikahisa S., Nishino S. (2011). Using iPSC-derived neurons to uncover cellular phenotypes associated with Timothy syndrome. Nat. Med..

[14] Fuzik J., Zeisel A., Mate Z., Calvigioni D., Yanagawa Y., Szabo G., Linnarsson S., Harkany T. (2016). Integration of electrophysiological recordings with single-cell RNA-seq data identifies neuronal subtypes. Nat. Biotechnol..

[15] Ono Y., Nakatani T., Sakamoto Y., Mizuhara E., Minaki Y., Kumai M., Hamaguchi A., Nishimura M., Inoue Y., Hayashi H. (2007). Differences in neurogenic potential in floor plate cells along an anteroposterior location: Midbrain dopaminergic neurons originate from mesencephalic floor plate cells. Development.

[16] Kim J.H., Auerbach J.M., Rodriguez-Gomez J.A., Velasco I., Gavin D., Lumelsky N., Lee S.H., Nguyen J., Sanchez-Pernaute R., Bankiewicz K. (2002). Dopamine neurons derived from embryonic stem cells function in an animal model of Parkinson’s disease. Nature.

[17] Chung S., Hedlund E., Hwang M., Kim D.W., Shin B.S., Hwang D.Y., Kang U.J., Isacson O., Kim K.S. (2005). The homeodomain transcription factor Pitx3 facilitates differentiation of mouse embryonic stem cells into AHD2-expressing dopaminergic neurons. Mol. Cell. Neurosci..

[18] Kim D.W., Chung S., Hwang M., Ferree A., Tsai H.C., Park J.J., Chung S., Nam T.S., Kang U.J., Isacson O. (2006). Stromal cell-derived inducing activity, Nurr1, and signaling molecules synergistically induce dopaminergic neurons from mouse embryonic stem cells. Stem Cells.

[19] Kriks S., Shim J.W., Piao J., Ganat Y.M., Wakeman D.R., Xie Z., Carrillo-Reid L., Auyeung G., Antonacci C., Buch A. (2011). Dopamine neurons derived from human ES cells efficiently engraft in animal models of Parkinson’s disease. Nature.

[20] Ryan S.D., Dolatabadi N., Chan S.F., Zhang X., Akhtar M.W., Parker J., Soldner F., Sunico C.R., Nagar S., Talantova M. (2013). Isogenic human iPSC Parkinson’s model shows nitrosative stress-induced dysfunction in MEF2-PGC1α transcription. Cell.

[21] Kirkeby A., Grealish S., Wolf D.A., Nelander J., Wood J., Lundblad M., Lindvall O., Parmar M. (2012). Generation of regionally specified neural progenitors and functional neurons from human embryonic stem cells under defined conditions. Cell Rep..

[22] Hyysalo A., Ristola M., Makinen M.E., Hayrynen S., Nykter M., Narkilahti S. (2017). Laminin α5 substrates promote survival, network formation and functional development of human pluripotent stem cell-derived neurons in vitro. Stem Cell Res..

[23] Ambasudhan R., Dolatabadi N., Nutter A., Masliah E., McKercher S.R., Lipton S.A. (2014). Potential for cell therapy in Parkinson’s disease using genetically programmed human embryonic stem cell-derived neural progenitor cells. J. Comp. Neurol..

[24] El-Akabawy G., Medina L.M., Jeffries A., Price J., Modo M. (2011). Purmorphamine increases DARPP-32 differentiation in human striatal neural stem cells through the Hedgehog pathway. Stem Cells Dev..

[25] Arber C., Precious S.V., Cambray S., Risner-Janiczek J.R., Kelly C., Noakes Z., Fjodorova M., Heuer A., Ungless M.A., Rodriguez T.A. (2015). Activin A directs striatal projection neuron differentiation of human pluripotent stem cells. Development.

[26] Ma L., Hu B., Liu Y., Vermilyea S.C., Liu H., Gao L., Sun Y., Zhang X., Zhang S.C. (2012). Human embryonic stem cell-derived GABA neurons correct locomotion deficits in quinolinic acid-lesioned mice. Cell Stem Cell.

[27] Lin L., Yuan J., Sander B., Golas M.M. (2015). In vitro differentiation of human neural progenitor cells into striatal GABAergic neurons. Stem Cells Transl. Med..

[28] Krencik R., Zhang S.C. (2011). Directed differentiation of functional astroglial subtypes from human pluripotent stem cells. Nat. Protoc..

[29] Santos R., Vadodaria K.C., Jaeger B.N., Mei A., Lefcochilos-Fogelquist S., Mendes A.P.D., Erikson G., Shokhirev M., Randolph-Moore L., Fredlender C. (2017). Differentiation of inflammation-responsive astrocytes from glial progenitors generated from human induced pluripotent stem cells. Stem Cell Rep..

[30] Palm T., Bolognin S., Meiser J., Nickels S., Trager C., Meilenbrock R.L., Brockhaus J., Schreitmuller M., Missler M., Schwamborn J.C. (2015). Rapid and robust generation of long-term self-renewing human neural stem cells with the ability to generate mature astroglia. Sci. Rep..

[31] Tcw J., Wang M., Pimenova A.A., Bowles K.R., Hartley B.J., Lacin E., Machlovi S.I., Abdelaal R., Karch C.M., Phatnani H. (2017). An efficient platform for astrocyte differentiation from human induced pluripotent stem cells. Stem Cell Rep..

[32] Canals I., Ginisty A., Quist E., Timmerman R., Fritze J., Miskinyte G., Monni E., Hansen M.G., Hidalgo I., Bryder D. (2018). Rapid and efficient induction of functional astrocytes from human pluripotent stem cells. Nat. Methods.

[33] Hickman S.E., Kingery N.D., Ohsumi T.K., Borowsky M.L., Wang L.C., Means T.K., El Khoury J. (2013). The microglial sensome revealed by direct RNA sequencing. Nat. Neurosci..

[34] Hollingworth P., Harold D., Sims R., Gerrish A., Lambert J.C., Carrasquillo M.M., Abraham R., Hamshere M.L., Pahwa J.S., Moskvina V. (2011). Common variants at ABCA7, MS4A6A/MS4A4E, EPHA1, CD33 and CD2AP are associated with Alzheimer’s disease. Nat. Genet..

[35] Heppner F.L., Ransohoff R.M., Becher B. (2015). Immune attack: The role of inflammation in Alzheimer disease. Nat. Rev. Neurosci..

[36] Colonna M., Wang Y. (2016). TREM2 variants: New keys to decipher Alzheimer disease pathogenesis. Nat. Rev. Neurosci..

[37] Webers A., Heneka M.T., Gleeson P.A. (2020). The role of innate immune responses and neuroinflammation in amyloid accumulation and progression of Alzheimer’s disease. Immunol. Cell Biol..

[38] Heneka M.T., Kummer M.P., Latz E. (2014). Innate immune activation in neurodegenerative disease. Nat. Rev. Immunol..

[39] Ouchi Y., Yoshikawa E., Sekine Y., Futatsubashi M., Kanno T., Ogusu T., Torizuka T. (2005). Microglial activation and dopamine terminal loss in early Parkinson’s disease. Ann. Neurol..

[40] Wong Y.C., Krainc D. (2017). α-synuclein toxicity in neurodegeneration: Mechanism and therapeutic strategies. Nat. Med..

[41] Ferreira S.A., Romero-Ramos M. (2018). Microglia response during parkinson’s disease: α-Synuclein intervention. Front. Cell. Neurosci..

[42] Geloso M.C., Corvino V., Marchese E., Serrano A., Michetti F., D’Ambrosi N. (2017). The dual role of microglia in ALS: Mechanisms and therapeutic approaches. Front. Aging Neurosci..

[43] Spiller K.J., Restrepo C.R., Khan T., Dominique M.A., Fang T.C., Canter R.G., Roberts C.J., Miller K.R., Ransohoff R.M., Trojanowski J.Q. (2018). Microglia-Mediated recovery from ALS-relevant motor neuron degeneration in a mouse model of TDP-43 proteinopathy. Nat. Neurosci..

[44] Palpagama T.H., Waldvogel H.J., Faull R.L.M., Kwakowsky A. (2019). The Role of Microglia and Astrocytes in Huntington’s Disease. Front. Mol. Neurosci..

[45] Abud E.M., Ramirez R.N., Martinez E.S., Healy L.M., Nguyen C.H.H., Newman S.A., Yeromin A.V., Scarfone V.M., Marsh S.E., Fimbres C. (2017). iPSC-derived human microglia-like cells to study neurological diseases. Neuron.

[46] Douvaras P., Sun B., Wang M., Kruglikov I., Lallos G., Zimmer M., Terrenoire C., Zhang B., Gandy S., Schadt E. (2017). Directed differentiation of human pluripotent stem cells to microglia. Stem Cell Rep..

[47] Muffat J., Li Y., Yuan B., Mitalipova M., Omer A., Corcoran S., Bakiasi G., Tsai L.H., Aubourg P., Ransohoff R.M. (2016). Efficient derivation of microglia-like cells from human pluripotent stem cells. Nat. Med..

[48] Pandya H., Shen M.J., Ichikawa D.M., Sedlock A.B., Choi Y., Johnson K.R., Kim G., Brown M.A., Elkahloun A.G., Maric D. (2017). Differentiation of human and murine induced pluripotent stem cells to microglia-like cells. Nat. Neurosci..

[49] Trudler D., Nazor K.L., Eisele Y.S., Grabauskas T., Dolatabadi N., Parker J., Sultan A., Zhong Z., Goodwin M.S., Levites Y. (2021). Soluble α-synuclein-antibody complexes activate the NLRP3 inflammasome in hiPSC-derived microglia. Proc. Natl. Acad. Sci. USA.

[50] Park J., Wetzel I., Marriott I., Dreau D., D’Avanzo C., Kim D.Y., Tanzi R.E., Cho H. (2018). A 3D human triculture system modeling neurodegeneration and neuroinflammation in Alzheimer’s disease. Nat. Neurosci..

[51] Choi S.H., Kim Y.H., Hebisch M., Sliwinski C., Lee S., D’Avanzo C., Chen H., Hooli B., Asselin C., Muffat J. (2014). A three-dimensional human neural cell culture model of Alzheimer’s disease. Nature.

[52] Sloan S.A., Andersen J., Pasca A.M., Birey F., Pasca S.P. (2018). Generation and assembly of human brain region-specific three-dimensional cultures. Nat. Protoc..

[53] Lancaster M.A., Renner M., Martin C.A., Wenzel D., Bicknell L.S., Hurles M.E., Homfray T., Penninger J.M., Jackson A.P., Knoblich J.A. (2013). Cerebral organoids model human brain development and microcephaly. Nature.

[54] Quadrato G., Nguyen T., Macosko E.Z., Sherwood J.L., Min Yang S., Berger D.R., Maria N., Scholvin J., Goldman M., Kinney J.P. (2017). Cell diversity and network dynamics in photosensitive human brain organoids. Nature.

[55] Sloan S.A., Darmanis S., Huber N., Khan T.A., Birey F., Caneda C., Reimer R., Quake S.R., Barres B.A., Pasca S.P. (2017). Human astrocyte maturation captured in 3D cerebral cortical spheroids derived from pluripotent stem cells. Neuron.

[56] Madhavan M., Nevin Z.S., Shick H.E., Garrison E., Clarkson-Paredes C., Karl M., Clayton B.L.L., Factor D.C., Allan K.C., Barbar L. (2018). Induction of myelinating oligodendrocytes in human cortical spheroids. Nat. Methods.

[57] Qian X., Nguyen H.N., Song M.M., Hadiono C., Ogden S.C., Hammack C., Yao B., Hamersky G.R., Jacob F., Zhong C. (2016). Brain-region-specific organoids using mini-bioreactors for modeling ZIKV exposure. Cell.

[58] Andersen J., Revah O., Miura Y., Thom N., Amin N.D., Kelley K.W., Singh M., Chen X., Thete M.V., Walczak E.M. (2020). Generation of functional human 3D cortico-motor assembloids. Cell.

[59] Miura Y., Li M.Y., Birey F., Ikeda K., Revah O., Thete M.V., Park J.Y., Puno A., Lee S.H., Porteus M.H. (2020). Generation of human striatal organoids and cortico-striatal assembloids from human pluripotent stem cells. Nat. Biotechnol..

[60] Zhao Z., Nelson A.R., Betsholtz C., Zlokovic B.V. (2015). Establishment and dysfunction of the blood-brain barrier. Cell.

[61] Mantle J.L., Lee K.H. (2018). A differentiating neural stem cell-derived astrocytic population mitigates the inflammatory effects of TNF-α and IL-6 in an iPSC-based blood-brain barrier model. Neurobiol. Dis..

[62] Canfield S.G., Stebbins M.J., Morales B.S., Asai S.W., Vatine G.D., Svendsen C.N., Palecek S.P., Shusta E.V. (2017). An isogenic blood-brain barrier model comprising brain endothelial cells, astrocytes, and neurons derived from human induced pluripotent stem cells. J. Neurochem..

[63] Pham M.T., Pollock K.M., Rose M.D., Cary W.A., Stewart H.R., Zhou P., Nolta J.A., Waldau B. (2018). Generation of human vascularized brain organoids. Neuroreport.

[64] Ham O., Jin Y.B., Kim J., Lee M.O. (2020). Blood vessel formation in cerebral organoids formed from human embryonic stem cells. Biochem. Biophys. Res. Commun..

[65] Mansour A.A., Goncalves J.T., Bloyd C.W., Li H., Fernandes S., Quang D., Johnston S., Parylak S.L., Jin X., Gage F.H. (2018). An in vivo model of functional and vascularized human brain organoids. Nat. Biotechnol..

[66] Aldewachi H., Al-Zidan R.N., Conner M.T., Salman M.M. (2021). High-throughput screening platforms in the discovery of novel drugs for neurodegenerative diseases. Bioengineering.

[67] Blay V., Tolani B., Ho S.P., Arkin M.R. (2020). High-Throughput Screening: Today’s biochemical and cell-based approaches. Drug Discov. Today.

[68] Janes J., Young M.E., Chen E., Rogers N.H., Burgstaller-Muehlbacher S., Hughes L.D., Love M.S., Hull M.V., Kuhen K.L., Woods A.K. (2018). The ReFRAME library as a comprehensive drug repurposing library and its application to the treatment of cryptosporidiosis. Proc. Natl. Acad. Sci. USA.

[69] Sirota M., Dudley J.T., Kim J., Chiang A.P., Morgan A.A., Sweet-Cordero A., Sage J., Butte A.J. (2011). Discovery and preclinical validation of drug indications using compendia of public gene expression data. Sci. Transl. Med..

[70] Van Noort V., Schölch S., Iskar M., Zeller G., Ostertag K., Schweitzer C., Werner K., Weitz J., Koch M., Bork P. (2014). Novel drug candidates for the treatment of metastatic colorectal cancer through global inverse gene-expression profiling. Cancer Res..

[71] Williams G., Gatt A., Clarke E., Corcoran J., Doherty P., Chambers D., Ballard C. (2019). Drug repurposing for Alzheimer’s disease based on transcriptional profiling of human iPSC-derived cortical neurons. Transl. Psychiatry.

[72] Lamb J., Crawford E.D., Peck D., Modell J.W., Blat I.C., Wrobel M.J., Lerner J., Brunet J.-P., Subramanian A., Ross K.N. (2006). The connectivity map: Using gene-expression signatures to connect small molecules, genes, and disease. Science.

[73] Subramanian A., Narayan R., Corsello S.M., Peck D.D., Natoli T.E., Lu X., Gould J., Davis J.F., Tubelli A.A., Asiedu J.K. (2017). A next generation connectivity map: L1000 platform and the first 1,000,000 profiles. Cell.

[74] Palop J.J., Mucke L. (2010). Amyloid-beta-induced neuronal dysfunction in Alzheimer’s disease: From synapses toward neural networks. Nat. Neurosci..

[75] Jellinger K.A., Bancher C. (1996). AD neuropathology. Neurology.

[76] Kondo T., Asai M., Tsukita K., Kutoku Y., Ohsawa Y., Sunada Y., Imamura K., Egawa N., Yahata N., Okita K. (2013). Modeling Alzheimer’s disease with iPSCs reveals stress phenotypes associated with intracellular Aβ and differential drug responsiveness. Cell Stem Cell.

[77] Muratore C.R., Rice H.C., Srikanth P., Callahan D.G., Shin T., Benjamin L.N., Walsh D.M., Selkoe D.J., Young-Pearse T.L. (2014). The familial Alzheimer’s disease APPV717I mutation alters APP processing and Tau expression in iPSC-derived neurons. Hum. Mol. Genet..

[78] Yagi T., Ito D., Okada Y., Akamatsu W., Nihei Y., Yoshizaki T., Yamanaka S., Okano H., Suzuki N. (2011). Modeling familial Alzheimer’s disease with induced pluripotent stem cells. Hum. Mol. Genet..

[79] Israel M.A., Yuan S.H., Bardy C., Reyna S.M., Mu Y., Herrera C., Hefferan M.P., van Gorp S., Nazor K.L., Boscolo F.S. (2012). Probing sporadic and familial Alzheimer’s disease using induced pluripotent stem cells. Nature.

[80] Oksanen M., Petersen A.J., Naumenko N., Puttonen K., Lehtonen S., Gubert Olive M., Shakirzyanova A., Leskela S., Sarajarvi T., Viitanen M. (2017). PSEN1 mutant iPSC-derived model reveals severe astrocyte pathology in Alzheimer’s disease. Stem Cell Rep..

[81] Liao M.C., Muratore C.R., Gierahn T.M., Sullivan S.E., Srikanth P., de Jager P.L., Love J.C., Young-Pearse T.L. (2016). Single-cell detection of secreted Aβ and sAPPα from human iPSC-derived neurons and astrocytes. J. Neurosci..

[82] Konttinen H., Cabral-da-Silva M.E.C., Ohtonen S., Wojciechowski S., Shakirzyanova A., Caligola S., Giugno R., Ishchenko Y., Hernandez D., Fazaludeen M.F. (2019). PSEN1DeltaE9, APPswe, and APOE4 confer disparate phenotypes in human iPSC-derived microglia. Stem Cell Rep..

[83] Ghatak S., Dolatabadi N., Trudler D., Zhang X., Wu Y., Mohata M., Ambasudhan R., Talantova M., Lipton S.A. (2019). Mechanisms of hyperexcitability in Alzheimer’s disease hiPSC-derived neurons and cerebral organoids vs. isogenic control. eLife.

[84] Ghatak S., Dolatabadi N., Gao R., Wu Y., Scott H., Trudler D., Sultan A., Ambasudhan R., Nakamura T., Masliah E. (2020). NitroSynapsin ameliorates hypersynchronous neural network activity in Alzheimer hiPSC models. Mol. Psychiatry.

[85] Kwart D., Gregg A., Scheckel C., Murphy E., Paquet D., Duffield M., Fak J., Olsen O., Darnell R., Tessier-Lavigne M. (2019). A large panel of isogenic APP and PSEN1 mutant human iPSC neurons reveals shared endosomal abnormalities mediated by APP β-CTFs, not Aβ. Neuron.

[86] Woodruff G., Young J.E., Martinez F.J., Buen F., Gore A., Kinaga J., Li Z., Yuan S.H., Zhang K., Goldstein L.S. (2013). The presenilin-1 DeltaE9 mutation results in reduced γ-secretase activity, but not total loss of PS1 function, in isogenic human stem cells. Cell Rep..

[87] Antonarakis S.E. (2017). Down syndrome and the complexity of genome dosage imbalance. Nat. Rev. Genet..

[88] Shi Y., Kirwan P., Smith J., MacLean G., Orkin S.H., Livesey F.J. (2012). A human stem cell model of early Alzheimer’s disease pathology in Down syndrome. Sci. Transl. Med..

[89] Chang C.-Y., Chen S.-M., Lu H.-E., Lai S.-M., Lai P.-S., Shen P.-W., Chen P.-Y., Shen C.-I., Harn H.-J., Lin S.-Z. (2015). N-butylidenephthalide attenuates Alzheimer’s disease-like cytopathy in down syndrome induced pluripotent stem cell-derived neurons. Sci. Rep..

[90] Dashinimaev E.B., Artyuhov A.S., Bolshakov A.P., Vorotelyak E.A., Vasiliev A.V. (2017). Neurons Derived from Induced Pluripotent Stem Cells of Patients with Down Syndrome Reproduce Early Stages of Alzheimer’s Disease Type Pathology in vitro. J. Alzheimer’s Dis..

[91] Ovchinnikov D.A., Korn O., Virshup I., Wells C.A., Wolvetang E.J. (2018). The impact of APP on Alzheimer-like pathogenesis and gene expression in down syndrome iPSC-derived neurons. Stem Cell Rep..

[92] Sposito T., Preza E., Mahoney C.J., Seto-Salvia N., Ryan N.S., Morris H.R., Arber C., Devine M.J., Houlden H., Warner T.T. (2015). Developmental regulation of tau splicing is disrupted in stem cell-derived neurons from frontotemporal dementia patients with the 10 + 16 splice-site mutation in MAPT. Hum. Mol. Genet..

[93] Sato C., Barthelemy N.R., Mawuenyega K.G., Patterson B.W., Gordon B.A., Jockel-Balsarotti J., Sullivan M., Crisp M.J., Kasten T., Kirmess K.M. (2018). Tau kinetics in neurons and the human central nervous system. Neuron.

[94] Yang J., Zhao H., Ma Y., Shi G., Song J., Tang Y., Li S., Li T., Liu N., Tang F. (2017). Early pathogenic event of Alzheimer’s disease documented in iPSCs from patients with PSEN1 mutations. Oncotarget.

[95] Choi S.H., Kim Y.H., D’Avanzo C., Aronson J., Tanzi R.E., Kim D.Y. (2015). Recapitulating amyloid β and tau pathology in human neural cell culture models: Clinical implications. US Neurol..

[96] Ochalek A., Mihalik B., Avci H.X., Chandrasekaran A., Teglasi A., Bock I., Giudice M.L., Tancos Z., Molnar K., Laszlo L. (2017). Neurons derived from sporadic Alzheimer’s disease iPSCs reveal elevated TAU hyperphosphorylation, increased amyloid levels, and GSK3B activation. Alzheimers Res. Ther..

[97] Lee H.K., Velazquez Sanchez C., Chen M., Morin P.J., Wells J.M., Hanlon E.B., Xia W. (2016). Three dimensional human neuro-spheroid model of Alzheimer’s disease based on differentiated induced pluripotent stem cells. PLoS ONE.

[98] Hossini A.M., Megges M., Prigione A., Lichtner B., Toliat M.R., Wruck W., Schroter F., Nuernberg P., Kroll H., Makrantonaki E. (2015). Induced pluripotent stem cell-derived neuronal cells from a sporadic Alzheimer’s disease donor as a model for investigating AD-associated gene regulatory networks. BMC Genom..

[99] Mertens J., Paquola A.C.M., Ku M., Hatch E., Bohnke L., Ladjevardi S., McGrath S., Campbell B., Lee H., Herdy J.R. (2015). Directly reprogrammed human neurons retain aging-associated transcriptomic signatures and reveal age-related nucleocytoplasmic defects. Cell Stem Cell.

[100] Lin Y.T., Seo J., Gao F., Feldman H.M., Wen H.L., Penney J., Cam H.P., Gjoneska E., Raja W.K., Cheng J. (2018). APOE4 causes widespread molecular and cellular alterations associated with Alzheimer’s disease phenotypes in human iPSC-derived brain cell types. Neuron.

[101] Zhao J., Davis M.D., Martens Y.A., Shinohara M., Graff-Radford N.R., Younkin S.G., Wszolek Z.K., Kanekiyo T., Bu G. (2017). APOE epsilon4/epsilon4 diminishes neurotrophic function of human iPSC-derived astrocytes. Hum. Mol. Genet..

[102] Wang C., Najm R., Xu Q., Jeong D.E., Walker D., Balestra M.E., Yoon S.Y., Yuan H., Li G., Miller Z.A. (2018). Gain of toxic apolipoprotein E4 effects in human iPSC-derived neurons is ameliorated by a small-molecule structure corrector. Nat. Med..

[103] Fernandez C.G., Hamby M.E., McReynolds M.L., Ray W.J. (2019). The role of APOE4 in disrupting the homeostatic functions of astrocytes and microglia in aging and Alzheimer’s disease. Front. Aging Neurosci..

[104] Mahley R.W., Huang Y. (2012). Apolipoprotein e sets the stage: Response to injury triggers neuropathology. Neuron.

[105] Parhizkar S., Arzberger T., Brendel M., Kleinberger G., Deussing M., Focke C., Nuscher B., Xiong M., Ghasemigharagoz A., Katzmarski N. (2019). Loss of TREM2 function increases amyloid seeding but reduces plaque-associated ApoE. Nat. Neurosci..

[106] Penney J., Ralvenius W.T., Tsai L.H. (2020). Modeling Alzheimer’s disease with iPSC-derived brain cells. Mol. Psychiatry.

[107] Kondo T., Imamura K., Funayama M., Tsukita K., Miyake M., Ohta A., Woltjen K., Nakagawa M., Asada T., Arai T. (2017). iPSC-based compound screening and in vitro trials identify a dynergistic snti-amyloid β combination for Alzheimer’s disease. Cell Rep..

[108] Jin M., O’Nuallain B., Hong W., Boyd J., Lagomarsino V.N., O’Malley T.T., Liu W., Vanderburg C.R., Frosch M.P., Young-Pearse T. (2018). An in vitro paradigm to assess potential anti-Aβ antibodies for Alzheimer’s disease. Nat. Commun..

[109] Cavazzoni P. FDA’s Decision to Approve New Treatment for Alzheimer’s Disease. https://www.fda.gov/drugs/news-events-human-drugs/fdas-decision-approve-new-treatment-alzheimers-disease.

[110] Park J.-C., Jang S.-Y., Lee D., Lee J., Kang U., Chang H., Kim H.J., Han S.-H., Seo J., Choi M. (2021). A logical network-based drug-screening platform for Alzheimer’s disease representing pathological features of human brain organoids. Nat. Commun..

[111] Gonzalez C., Armijo E., Bravo-Alegria J., Becerra-Calixto A., Mays C.E., Soto C. (2018). Modeling amyloid β and tau pathology in human cerebral organoids. Mol. Psychiatry.

[112] Kim Y.H., Choi S.H., D’Avanzo C., Hebisch M., Sliwinski C., Bylykbashi E., Washicosky K.J., Klee J.B., Brustle O., Tanzi R.E. (2015). A 3D human neural cell culture system for modeling Alzheimer’s disease. Nat. Protoc..

[113] Mertens J., Stuber K., Wunderlich P., Ladewig J., Kesavan J.C., Vandenberghe R., Vandenbulcke M., van Damme P., Walter J., Brustle O. (2013). APP processing in human pluripotent stem cell-derived neurons is resistant to NSAID-based γ-secretase modulation. Stem Cell Rep..

[114] Talantova M., Sanz-Blasco S., Zhang X., Xia P., Akhtar M.W., Okamoto S., Dziewczapolski G., Nakamura T., Cao G., Pratt A.E. (2013). Aβ induces astrocytic glutamate release, extrasynaptic NMDA receptor activation, and synaptic loss. Proc. Natl. Acad. Sci. USA.

[115] Wang M., Li A., Sekiya M., Beckmann N.D., Quan X., Schrode N., Fernando M.B., Yu A., Zhu L., Cao J. (2021). Transformative network modeling of multi-omics data reveals detailed circuits, key regulators, and potential therapeutics for Alzheimer’s disease. Neuron.

[116] Biundo R., Weis L., Antonini A. (2016). Cognitive decline in Parkinson’s disease: The complex picture. NPJ Parkinsons Dis..

[117] Diederich N.J., Fenelon G., Stebbins G., Goetz C.G. (2009). Hallucinations in Parkinson disease. Nat. Rev. Neurol..

[118] De Lau L.M., Breteler M.M. (2006). Epidemiology of Parkinson’s disease. Lancet Neurol..

[119] Trevor A.J., Castagnoli N., Singer T.P. (1988). The formation of reactive intermediates in the MAO-catalyzed oxidation of the nigrostriatal toxin 1-methyl-4-phenyl-1,2,3,6-tetrahydropyridine (MPTP). Toxicology.

[120] Ungerstedt U. (1968). 6-Hydroxy-dopamine induced degeneration of central monoamine neurons. Eur. J. Pharmacol..

[121] Xiong N., Long X., Xiong J., Jia M., Chen C., Huang J., Ghoorah D., Kong X., Lin Z., Wang T. (2012). Mitochondrial complex I inhibitor rotenone-induced toxicity and its potential mechanisms in Parkinson’s disease models. Crit. Rev. Toxicol..

[122] Blandini F., Armentero M.-T., Martignoni E. (2008). The 6-hydroxydopamine model: News from the past. Parkinsonism Relat. Disord..

[123] Tran J., Anastacio H., Bardy C. (2020). Genetic predispositions of Parkinson’s disease revealed in patient-derived brain cells. NPJ Parkinson’s Dis..

[124] Morais V.A., Haddad D., Craessaerts K., de Bock P.J., Swerts J., Vilain S., Aerts L., Overbergh L., Grunewald A., Seibler P. (2014). PINK1 loss-of-function mutations affect mitochondrial complex I activity via NdufA10 ubiquinone uncoupling. Science.

[125] Lachenmayer M.L., Yue Z. (2012). Genetic animal models for evaluating the role of autophagy in etiopathogenesis of Parkinson disease. Autophagy.

[126] Joselin A.P., Hewitt S.J., Callaghan S.M., Kim R.H., Chung Y.H., Mak T.W., Shen J., Slack R.S., Park D.S. (2012). ROS-dependent regulation of Parkin and DJ-1 localization during oxidative stress in neurons. Hum. Mol. Genet..

[127] Dawson T.M., Ko H.S., Dawson V.L. (2010). Genetic animal models of Parkinson’s disease. Neuron.

[128] Jenner P. (2015). Treatment of the later stages of Parkinson’s disease—Pharmacological approaches now and in the future. Transl. Neurodegener..

[129] Fahn S., Oakes D., Shoulson I., Kieburtz K., Rudolph A., Lang A., Olanow C.W., Tanner C., Marek K., Parkinson Study Group (2004). Levodopa and the progression of Parkinson’s disease. N. Engl. J. Med..

[130] Olanow C.W., Rascol O., Hauser R., Feigin P.D., Jankovic J., Lang A., Langston W., Melamed E., Poewe W., Stocchi F. (2009). A double-blind, delayed-start trial of rasagiline in Parkinson’s disease. N. Engl. J. Med..

[131] Piao J., Zabierowski S., Dubose B.N., Hill E.J., Navare M., Claros N., Rosen S., Ramnarine K., Horn C., Fredrickson C. (2021). Preclinical efficacy and safety of a human embryonic stem cell-derived midbrain dopamine progenitor product, MSK-DA01. Cell Stem Cell.

[132] Soldner F., Laganiere J., Cheng A.W., Hockemeyer D., Gao Q., Alagappan R., Khurana V., Golbe L.I., Myers R.H., Lindquist S. (2011). Generation of isogenic pluripotent stem cells differing exclusively at two early onset Parkinson point mutations. Cell.

[133] Devine M.J., Ryten M., Vodicka P., Thomson A.J., Burdon T., Houlden H., Cavaleri F., Nagano M., Drummond N.J., Taanman J.W. (2011). Parkinson’s disease induced pluripotent stem cells with triplication of the α-synuclein locus. Nat. Commun..

[134] Byers B., Cord B., Nguyen H.N., Schüle B., Fenno L., Lee P.C., Deisseroth K., Langston J.W., Pera R.R., Palmer T.D. (2011). SNCA triplication Parkinson’s patient’s iPSC-derived DA neurons accumulate α-synuclein and are susceptible to oxidative stress. PLoS ONE.

[135] Oliveira L.M., Falomir-Lockhart L.J., Botelho M.G., Lin K.H., Wales P., Koch J.C., Gerhardt E., Taschenberger H., Outeiro T.F., Lingor P. (2015). Elevated α-synuclein caused by SNCA gene triplication impairs neuronal differentiation and maturation in Parkinson’s patient-derived induced pluripotent stem cells. Cell Death Dis..

[136] Flierl A., Oliveira L.M.A., Falomir-Lockhart L.J., Mak S.K., Hesley J., Soldner F., Arndt-Jovin D.J., Jaenisch R., Langston J.W., Jovin T.M. (2014). Higher vulnerability and stress sensitivity of neuronal precursor cells carrying an α-csnuclein gene triplication. PLoS ONE.

[137] Thakur P., Breger L.S., Lundblad M., Wan O.W., Mattsson B., Luk K.C., Lee V.M.Y., Trojanowski J.Q., Bjorklund A. (2017). Modeling Parkinson’s disease pathology by combination of fibril seeds and α-synuclein overexpression in the rat brain. Proc. Natl. Acad. Sci. USA.

[138] Wu Q., Takano H., Riddle D.M., Trojanowski J.Q., Coulter D.A., Lee V.M. (2019). Alpha-Synuclein (alphaSyn) Preformed Fibrils Induce Endogenous alphaSyn Aggregation, Compromise Synaptic Activity and Enhance Synapse Loss in Cultured Excitatory Hippocampal Neurons. J. Neurosci..

[139] Gribaudo S., Tixador P., Bousset L., Fenyi A., Lino P., Melki R., Peyrin J.M., Perrier A.L. (2019). Propagation of alpha-Synuclein Strains within Human Reconstructed Neuronal Network. Stem Cell Rep..

[140] Prots I., Grosch J., Brazdis R.M., Simmnacher K., Veber V., Havlicek S., Hannappel C., Krach F., Krumbiegel M., Schütz O. (2018). α-Synuclein oligomers induce early axonal dysfunction in human iPSC-based models of synucleinopathies. Proc. Natl. Acad. Sci. USA.

[141] Trudler D., Sanz-Blasco S., Eisele Y.S., Ghatak S., Bodhinathan K., Akhtar M.W., Lynch W.P., Pina-Crespo J.C., Talantova M., Kelly J.W. (2021). α-Synuclein Oligomers Induce Glutamate Release from Astrocytes and Excessive Extrasynaptic NMDAR Activity in Neurons, Thus Contributing to Synapse Loss. J. Neurosci..

[142] Nuytemans K., Theuns J., Cruts M., van Broeckhoven C. (2010). Genetic etiology of Parkinson disease associated with mutations in the SNCA, PARK2, PINK1, PARK7, and LRRK2 genes: A mutation update. Hum. Mutat..

[143] Klein C., Westenberger A. (2012). Genetics of Parkinson’s disease. Cold Spring Harb. Perspect. Med..

[144] West A.B., Moore D.J., Biskup S., Bugayenko A., Smith W.W., Ross C.A., Dawson V.L., Dawson T.M. (2005). Parkinson’s disease-associated mutations in leucine-rich repeat kinase 2 augment kinase activity. Proc. Natl. Acad. Sci. USA.

[145] Nguyen H.N., Byers B., Cord B., Shcheglovitov A., Byrne J., Gujar P., Kee K., Schule B., Dolmetsch R.E., Langston W. (2011). LRRK2 mutant iPSC-derived DA neurons demonstrate increased susceptibility to oxidative stress. Cell Stem Cell.

[146] Sanders L.H., Laganiere J., Cooper O., Mak S.K., Vu B.J., Huang Y.A., Paschon D.E., Vangipuram M., Sundararajan R., Urnov F.D. (2014). LRRK2 mutations cause mitochondrial DNA damage in iPSC-derived neural cells from Parkinson’s disease patients: Reversal by gene correction. Neurobiol. Dis..

[147] Reinhardt P., Schmid B., Burbulla L.F., Schondorf D.C., Wagner L., Glatza M., Hoing S., Hargus G., Heck S.A., Dhingra A. (2013). Genetic correction of a LRRK2 mutation in human iPSCs links parkinsonian neurodegeneration to ERK-dependent changes in gene expression. Cell Stem Cell.

[148] Qing X., Walter J., Jarazo J., Arias-Fuenzalida J., Hillje A.L., Schwamborn J.C. (2017). CRISPR/Cas9 and piggyBac-mediated footprint-free LRRK2-G2019S knock-in reveals neuronal complexity phenotypes and α-Synuclein modulation in dopaminergic neurons. Stem Cell Res..

[149] Carrion M.D.P., Marsicano S., Daniele F., Marte A., Pischedda F., Di Cairano E., Piovesana E., von Zweydorf F., Kremmer E., Gloeckner C.J. (2017). The LRRK2 G2385R variant is a partial loss-of-function mutation that affects synaptic vesicle trafficking through altered protein interactions. Sci. Rep..

[150] Ma D., Ng E.Y., Zeng L., Lim C.Y., Zhao Y., Tan E.K. (2017). Development of a human induced pluripotent stem cell (iPSC) line from a Parkinson’s disease patient carrying the N551K variant in LRRK2 gene. Stem Cell Res..

[151] Ma D., Zhou W., Ng E.Y., Zeng L., Zhao Y., Tan E.K. (2017). Reprogramming of a human induced pluripotent stem cell (iPSC) line from a Parkinson’s disease patient with a R1628P variant in the LRRK2 gene. Stem Cell Res..

[152] Ren Y., Jiang H., Hu Z., Fan K., Wang J., Janoschka S., Wang X., Ge S., Feng J. (2015). Parkin mutations reduce the complexity of neuronal processes in iPSC-derived human neurons. Stem Cells.

[153] Chung S.Y., Kishinevsky S., Mazzulli J.R., Graziotto J., Mrejeru A., Mosharov E.V., Puspita L., Valiulahi P., Sulzer D., Milner T.A. (2016). Parkin and PINK1 patient iPSC-derived midbrain dopamine neurons exhibit mitochondrial dysfunction and α-Synuclein accumulation. Stem Cell Rep..

[154] Cooper O., Seo H., Andrabi S., Guardia-Laguarta C., Graziotto J., Sundberg M., McLean J.R., Carrillo-Reid L., Xie Z., Osborn T. (2012). Pharmacological rescue of mitochondrial deficits in iPSC-derived neural cells from patients with familial Parkinson’s disease. Sci. Transl. Med..

[155] Oh C.K., Sultan A., Platzer J., Dolatabadi N., Soldner F., McClatchy D.B., Diedrich J.K., Yates J.R., Ambasudhan R., Nakamura T. (2017). S-Nitrosylation of PINK1 attenuates PINK1/Parkin-eependent mitophagy in hiPSC-based parkinson’s disease models. Cell Rep..

[156] Panagiotakopoulou V., Ivanyuk D., de Cicco S., Haq W., Arsić A., Yu C., Messelodi D., Oldrati M., Schöndorf D.C., Perez M.-J. (2020). Interferon-γ signaling synergizes with LRRK2 in neurons and microglia derived from human induced pluripotent stem cells. Nat. Commun..

[157] Iannielli A., Bido S., Folladori L., Segnali A., Cancellieri C., Maresca A., Massimino L., Rubio A., Morabito G., Caporali L. (2018). Pharmacological inhibition of necroptosis protects from dopaminergic neuronal cell death in parkinson’s disease models. Cell Rep..

[158] Sanchez-Danes A., Richaud-Patin Y., Carballo-Carbajal I., Jimenez-Delgado S., Caig C., Mora S., Di Guglielmo C., Ezquerra M., Patel B., Giralt A. (2012). Disease-specific phenotypes in dopamine neurons from human iPS-based models of genetic and sporadic Parkinson’s disease. EMBO Mol. Med..

[159] Mazzulli J.R., Zunke F., Isacson O., Studer L., Krainc D. (2016). α-Synuclein-induced lysosomal dysfunction occurs through disruptions in protein trafficking in human midbrain synucleinopathy models. Proc. Natl. Acad. Sci. USA.

[160] Fernández-Santiago R., Carballo-Carbajal I., Castellano G., Torrent R., Richaud Y., Sánchez-Danés A., Vilarrasa-Blasi R., Sánchez-Pla A., Mosquera J.L., Soriano J. (2015). Aberrant epigenome in iPSC-derived dopaminergic neurons from Parkinson’s disease patients. EMBO Mol. Med..

[161] Fernandez-Santiago R., Merkel A., Castellano G., Heath S., Raya A., Tolosa E., Marti M.J., Consiglio A., Ezquerra M. (2019). Whole-genome DNA hyper-methylation in iPSC-derived dopaminergic neurons from Parkinson’s disease patients. Clin. Epigenet..

[162] Schulze M., Sommer A., Plötz S., Farrell M., Winner B., Grosch J., Winkler J., Riemenschneider M.J. (2018). Sporadic Parkinson’s disease derived neuronal cells show disease-specific mRNA and small RNA signatures with abundant deregulation of piRNAs. Acta Neuropathol. Commun..

[163] Tabata Y., Imaizumi Y., Sugawara M., Andoh-Noda T., Banno S., Chai M., Sone T., Yamazaki K., Ito M., Tsukahara K. (2018). T-type calcium channels determine the vulnerability of dopaminergic neurons to mitochondrial stress in familial parkinson disease. Stem Cell Rep..

[164] Yamaguchi A., Ishikawa K.I., Inoshita T., Shiba-Fukushima K., Saiki S., Hatano T., Mori A., Oji Y., Okuzumi A., Li Y. (2020). Identifying therapeutic agents for amelioration of mitochondrial clearance disorder in neurons of familial Parkinson disease. Stem Cell Rep..

[165] Antoniou N., Prodromidou K., Kouroupi G., Samiotaki M., Panayotou G., Xilouri M., Stefanis L., Grailhe R., Taoufik E., Matsas R. (2020). High content screening and proteomic analysis identify the kinase Inhibitor BX795 as a potent neuroprotective compound in a patient-derived model of parkinson’s disease. bioRxiv.

[166] Kefalopoulou Z., Politis M., Piccini P., Mencacci N., Bhatia K., Jahanshahi M., Widner H., Rehncrona S., Brundin P., Björklund A. (2014). Long-term Clinical Outcome of Fetal Cell Transplantation for Parkinson Disease: Two Case Reports. JAMA Neurol..

[167] Kikuchi T., Morizane A., Doi D., Magotani H., Onoe H., Hayashi T., Mizuma H., Takara S., Takahashi R., Inoue H. (2017). Human iPS cell-derived dopaminergic neurons function in a primate Parkinson’s disease model. Nature.

[168] Morizane A., Kikuchi T., Hayashi T., Mizuma H., Takara S., Doi H., Mawatari A., Glasser M.F., Shiina T., Ishigaki H. (2017). MHC matching improves engraftment of iPSC-derived neurons in non-human primates. Nat. Commun..

[169] Mejzini R., Flynn L.L., Pitout I.L., Fletcher S., Wilton S.D., Akkari P.A. (2019). ALS Genetics, Mechanisms, and Therapeutics: Where Are We Now?. Front. Neurosci..

[170] Kim G., Gautier O., Tassoni-Tsuchida E., Ma X.R., Gitler A.D. (2020). ALS Genetics: Gains, Losses, and Implications for Future Therapies. Neuron.

[171] Serio A., Patani R. (2018). Concise Review: The Cellular Conspiracy of Amyotrophic Lateral Sclerosis. Stem Cells.

[172] Chen H., Qian K., Du Z., Cao J., Petersen A., Liu H., Blackbourn L.W.T., Huang C.L., Errigo A., Yin Y. (2014). Modeling ALS with iPSCs reveals that mutant SOD1 misregulates neurofilament balance in motor neurons. Cell Stem Cell.

[173] Kiskinis E., Sandoe J., Williams L.A., Boulting G.L., Moccia R., Wainger B.J., Han S., Peng T., Thams S., Mikkilineni S. (2014). Pathways disrupted in human ALS motor neurons identified through genetic correction of mutant SOD1. Cell Stem Cell.

[174] Wainger B.J., Kiskinis E., Mellin C., Wiskow O., Han S.S., Sandoe J., Perez N.P., Williams L.A., Lee S., Boulting G. (2014). Intrinsic membrane hyperexcitability of amyotrophic lateral sclerosis patient-derived motor neurons. Cell Rep..

[175] Kim B.W., Ryu J., Jeong Y.E., Kim J., Martin L.J. (2020). Human Motor Neurons With SOD1-G93A Mutation Generated From CRISPR/Cas9 Gene-Edited iPSCs Develop Pathological Features of Amyotrophic Lateral Sclerosis. Front. Cell Neurosci..

[176] Tyzack G.E., Hall C.E., Sibley C.R., Cymes T., Forostyak S., Carlino G., Meyer I.F., Schiavo G., Zhang S.-C., Gibbons G.M. (2017). A neuroprotective astrocyte state is induced by neuronal signal EphB1 but fails in ALS models. Nat. Commun..

[177] Kelley K.W., Ben Haim L., Schirmer L., Tyzack G.E., Tolman M., Miller J.G., Tsai H.-H., Chang S.M., Molofsky A.V., Yang Y. (2018). Kir4.1-dependent astrocyte-fast motor neuron interactions wre required for peak strength. Neuron.

[178] Egawa N., Kitaoka S., Tsukita K., Naitoh M., Takahashi K., Yamamoto T., Adachi F., Kondo T., Okita K., Asaka I. (2012). Drug screening for ALS using patient-specific induced pluripotent stem cells. Sci. Transl. Med..

[179] Devlin A.C., Burr K., Borooah S., Foster J.D., Cleary E.M., Geti I., Vallier L., Shaw C.E., Chandran S., Miles G.B. (2015). Human iPSC-derived motoneurons harbouring TARDBP or C9ORF72 ALS mutations are dysfunctional despite maintaining viability. Nat. Commun..

[180] Serio A., Bilican B., Barmada S.J., Ando D.M., Zhao C., Siller R., Burr K., Haghi G., Story D., Nishimura A.L. (2013). Astrocyte pathology and the absence of non-cell autonomy in an induced pluripotent stem cell model of TDP-43 proteinopathy. Proc. Natl. Acad. Sci. USA.

[181] Zhang Z., Almeida S., Lu Y., Nishimura A.L., Peng L., Sun D., Wu B., Karydas A.M., Tartaglia M.C., Fong J.C. (2013). Downregulation of microRNA-9 in iPSC-derived neurons of FTD/ALS patients with TDP-43 mutations. PLoS ONE.

[182] Naujock M., Stanslowsky N., Bufler S., Naumann M., Reinhardt P., Sterneckert J., Kefalakes E., Kassebaum C., Bursch F., Lojewski X. (2016). 4-Aminopyridine induced activity rescues hypoexcitable motor neurons from amyotrophic lateral sclerosis patient-derived induced pluripotent stem cells. Stem Cells.

[183] Liu M.L., Zang T., Zhang C.L. (2016). Direct lineage reprogramming reveals disease-specific phenotypes of motor neurons from human ALS patients. Cell Rep..

[184] Higelin J., Demestre M., Putz S., Delling J.P., Jacob C., Lutz A.K., Bausinger J., Huber A.K., Klingenstein M., Barbi G. (2016). FUS mislocalization and vulnerability to DNA damage in ALS patients derived hiPSCs and aging motoneurons. Front. Cell. Neurosci..

[185] Guo W., Naujock M., Fumagalli L., Vandoorne T., Baatsen P., Boon R., Ordovás L., Patel A., Welters M., Vanwelden T. (2017). HDAC6 inhibition reverses axonal transport defects in motor neurons derived from FUS-ALS patients. Nat. Commun..

[186] Donnelly C.J., Zhang P.W., Pham J.T., Haeusler A.R., Mistry N.A., Vidensky S., Daley E.L., Poth E.M., Hoover B., Fines D.M. (2013). RNA toxicity from the ALS/FTD C9ORF72 expansion is mitigated by antisense intervention. Neuron.

[187] Sareen D., O’Rourke J.G., Meera P., Muhammad A.K., Grant S., Simpkinson M., Bell S., Carmona S., Ornelas L., Sahabian A. (2013). Targeting RNA foci in iPSC-derived motor neurons from ALS patients with a C9ORF72 repeat expansion. Sci. Transl. Med..

[188] Westergard T., Jensen B.K., Wen X., Cai J., Kropf E., Iacovitti L., Pasinelli P., Trotti D. (2016). Cell-to-cell transmission of dipeptide repeat proteins linked to C9orf72-ALS/FTD. Cell Rep..

[189] Trageser K.J., Smith C., Herman F.J., Ono K., Pasinetti G.M. (2019). Mechanisms of Immune Activation by c9orf72-Expansions in Amyotrophic Lateral Sclerosis and Frontotemporal Dementia. Front. Neurosci..

[190] Dafinca R., Barbagallo P., Farrimond L., Candalija A., Scaber J., Ababneh N.A.A., Sathyaprakash C., Vowles J., Cowley S.A., Talbot K. (2020). Impairment of mitochondrial calcium buffering links mutations in C9ORF72 and TARDBP in iPS-derived motor neurons from patients with ALS/FTD. Stem Cell Rep..

[191] Dafinca R., Scaber J., Ababneh N., Lalic T., Weir G., Christian H., Vowles J., Douglas A.G., Fletcher-Jones A., Browne C. (2016). C9orf72 hexanucleotide expansions are associated with altered endoplasmic reticulum calcium homeostasis and stress granule formation in induced pluripotent stem cell-derived neurons from patients with amyotrophic lateral sclerosis and frontotemporal dementia. Stem Cells.

[192] Lopez-Gonzalez R., Lu Y., Gendron T.F., Karydas A., Tran H., Yang D., Petrucelli L., Miller B.L., Almeida S., Gao F.B. (2016). Poly(GR) in C9ORF72-related ALS/FTD compromises mitochondrial function and increases oxidative stress and DNA damage in iPSC-derived motor neurons. Neuron.

[193] Almeida S., Gascon E., Tran H., Chou H.J., Gendron T.F., Degroot S., Tapper A.R., Sellier C., Charlet-Berguerand N., Karydas A. (2013). Modeling key pathological features of frontotemporal dementia with C9ORF72 repeat expansion in iPSC-derived human neurons. Acta Neuropathol..

[194] Mackenzie I.R., Frick P., Neumann M. (2014). The neuropathology associated with repeat expansions in the C9ORF72 gene. Acta Neuropathol..

[195] Webster C.P., Smith E.F., Grierson A.J., de Vos K.J. (2018). C9orf72 plays a central role in Rab GTPase-dependent regulation of autophagy. Small GTPases.

[196] Webster C.P., Smith E.F., Bauer C.S., Moller A., Hautbergue G.M., Ferraiuolo L., Myszczynska M.A., Higginbottom A., Walsh M.J., Whitworth A.J. (2016). The C9orf72 protein interacts with Rab1a and the ULK1 complex to regulate initiation of autophagy. EMBO J..

[197] Freibaum B.D., Lu Y., Lopez-Gonzalez R., Kim N.C., Almeida S., Lee K.H., Badders N., Valentine M., Miller B.L., Wong P.C. (2015). GGGGCC repeat expansion in C9orf72 compromises nucleocytoplasmic transport. Nature.

[198] Zhang K., Donnelly C.J., Haeusler A.R., Grima J.C., Machamer J.B., Steinwald P., Daley E.L., Miller S.J., Cunningham K.M., Vidensky S. (2015). The C9orf72 repeat expansion disrupts nucleocytoplasmic transport. Nature.

[199] Meyer K., Ferraiuolo L., Miranda C.J., Likhite S., McElroy S., Renusch S., Ditsworth D., Lagier-Tourenne C., Smith R.A., Ravits J. (2014). Direct conversion of patient fibroblasts demonstrates non-cell autonomous toxicity of astrocytes to motor neurons in familial and sporadic ALS. Proc. Natl. Acad. Sci. USA.

[200] Birger A., Ben-Dor I., Ottolenghi M., Turetsky T., Gil Y., Sweetat S., Perez L., Belzer V., Casden N., Steiner D. (2019). Human iPSC-derived astrocytes from ALS patients with mutated C9ORF72 show increased oxidative stress and neurotoxicity. EBioMedicine.

[201] Ferraiuolo L., Meyer K., Sherwood T.W., Vick J., Likhite S., Frakes A., Miranda C.J., Braun L., Heath P.R., Pineda R. (2016). Oligodendrocytes contribute to motor neuron death in ALS via SOD1-dependent mechanism. Proc. Natl. Acad. Sci. USA.

[202] Lorenzini I., Alsop E., Levy J., Gittings L.M., Rabichow B.E., Lall D., Moore S., Bustos L., Pevey R., Burciu C. (2020). Activated iPSC-microglia from C9orf72 ALS/FTD patients exhibit endosomal-lysosomal dysfunction. bioRxiv.

[203] Burkhardt M.F., Martinez F.J., Wright S., Ramos C., Volfson D., Mason M., Garnes J., Dang V., Lievers J., Shoukat-Mumtaz U. (2013). A cellular model for sporadic ALS using patient-derived induced pluripotent stem cells. Mol. Cell. Neurosci..

[204] Alves C.J., Dariolli R., Jorge F.M., Monteiro M.R., Maximino J.R., Martins R.S., Strauss B.E., Krieger J.E., Callegaro D., Chadi G. (2015). Gene expression profiling for human iPS-derived motor neurons from sporadic ALS patients reveals a strong association between mitochondrial functions and neurodegeneration. Front. Cell. Neurosci..

[205] Imamura K., Izumi Y., Watanabe A., Tsukita K., Woltjen K., Yamamoto T., Hotta A., Kondo T., Kitaoka S., Ohta A. (2017). The Src/c-Abl pathway is a potential therapeutic target in amyotrophic lateral sclerosis. Sci. Transl. Med..

[206] Fujimori K., Ishikawa M., Otomo A., Atsuta N., Nakamura R., Akiyama T., Hadano S., Aoki M., Saya H., Sobue G. (2018). Modeling sporadic ALS in iPSC-derived motor neurons identifies a potential therapeutic agent. Nat. Med..

[207] Noto Y., Shibuya K., Vucic S., Kiernan M.C. (2016). Novel therapies in development that inhibit motor neuron hyperexcitability in amyotrophic lateral sclerosis. Expert Rev. Neurother..

[208] Kovalchuk M.O., Heuberger J., Sleutjes B., Ziagkos D., van den Berg L.H., Ferguson T.A., Franssen H., Groeneveld G.J. (2018). Acute effects of Riluzole and Retigabine on axonal excitability in patients with Amyotrophic lateral sclerosis: A randomized, double-blind, placebo-controlled, crossover trial. Clin. Pharmacol. Ther..

[209] De Boer A.S., Eggan K. (2015). A perspective on stem cell modeling of amyotrophic lateral sclerosis. Cell Cycle.

[210] Huang X., Roet K.C.D., Zhang L., Brault A., Berg A.P., Jefferson A.B., Klug-McLeod J., Leach K.L., Vincent F., Yang H. (2021). Human amyotrophic lateral sclerosis excitability phenotype screen: Target discovery and validation. Cell Rep..

[211] Volpato V., Webber C. (2020). Addressing variability in iPSC-derived models of human disease: Guidelines to promote reproducibility. Dis. Model. Mech..

[212] Lancaster M.A., Knoblich J.A. (2014). Generation of cerebral organoids from human pluripotent stem cells. Nat. Protoc..

[213] Yoon S.J., Elahi L.S., Pașca A.M., Marton R.M., Gordon A., Revah O., Miura Y., Walczak E.M., Holdgate G.M., Fan H.C. (2019). Reliability of human cortical organoid generation. Nat. Methods.

[214] Velasco S., Kedaigle A.J., Simmons S.K., Nash A., Rocha M., Quadrato G., Paulsen B., Nguyen L., Adiconis X., Regev A. (2019). Individual brain organoids reproducibly form cell diversity of the human cerebral cortex. Nature.

[215] Velasco V., Shariati S.A., Esfandyarpour R. (2020). Microtechnology-based methods for organoid models. Microsyst. Nanoeng..

[216] Carlson A.L., Bennett N.K., Francis N.L., Halikere A., Clarke S., Moore J.C., Hart R.P., Paradiso K., Wernig M., Kohn J. (2016). Generation and transplantation of reprogrammed human neurons in the brain using 3D microtopographic scaffolds. Nat. Commun..

[217] McMurtrey R.J. (2014). Patterned and functionalized nanofiber scaffolds in three-dimensional hydrogel constructs enhance neurite outgrowth and directional control. J. Neural Eng..

[218] Carballo-Molina O.A., Sánchez-Navarro A., López-Ornelas A., Lara-Rodarte R., Salazar P., Campos-Romo A., Ramos-Mejía V., Velasco I. (2016). Semaphorin 3C released from a biocompatible hydrogel guides and promotes axonal growth of rodent and human dopaminergic neurons. Tissue Eng. Part A.

[219] Lancaster M.A., Corsini N.S., Wolfinger S., Gustafson E.H., Phillips A.W., Burkard T.R., Otani T., Livesey F.J., Knoblich J.A. (2017). Guided self-organization and cortical plate formation in human brain organoids. Nat. Biotechnol..

[220] Oksdath M., Perrin S.L., Bardy C., Hilder E.F., DeForest C.A., Arrua R.D., Gomez G.A. (2018). Review: Synthetic scaffolds to control the biochemical, mechanical, and geometrical environment of stem cell-derived brain organoids. APL Bioeng..

[221] George J., Hsu C.-C., Nguyen L.T.B., Ye H., Cui Z. (2020). Neural tissue engineering with structured hydrogels in CNS models and therapies. Biotechnol. Adv..

[222] Svoboda D.S., Barrasa M.I., Shu J., Rietjens R., Zhang S., Mitalipova M., Berube P., Fu D., Shultz L.D., Bell G.W. (2019). Human iPSC-derived microglia assume a primary microglia-like state after transplantation into the neonatal mouse brain. Proc. Natl. Acad. Sci. USA.

[223] D’Alessio R., Koukouli F., Blanchard S., Catteau J., Raïs C., Lemonnier T., Féraud O., Bennaceur-Griscelli A., Groszer M., Maskos U. (2020). Long-term development of human iPSC-derived pyramidal neurons quantified after transplantation into the neonatal mouse cortex. Dev. Biol..

[224] Real R., Peter M., Trabalza A., Khan S., Smith M.A., Dopp J., Barnes S.J., Momoh A., Strano A., Volpi E. (2018). In vivo modeling of human neuron dynamics and Down syndrome. Science.

[225] Soldner F., Jaenisch R. (2018). Stem cells, genome editing, and the path to translational medicine. Cell.

[226] Okuno H., Nakabayashi K., Abe K., Ando T., Sanosaka T., Kohyama J., Akamatsu W., Ohyama M., Takahashi T., Kosaki K. (2017). Changeability of the fully methylated status of the 15q11.2 region in induced pluripotent stem cells derived from a patient with Prader-Willi syndrome. Congenit. Anom..

[227] Kim K., Doi A., Wen B., Ng K., Zhao R., Cahan P., Kim J., Aryee M.J., Ji H., Ehrlich L.I.R. (2010). Epigenetic memory in induced pluripotent stem cells. Nature.

[228] Vierbuchen T., Ostermeier A., Pang Z.P., Kokubu Y., Sudhof T.C., Wernig M. (2010). Direct conversion of fibroblasts to functional neurons by defined factors. Nature.

[229] Ambasudhan R., Talantova M., Coleman R., Yuan X., Zhu S., Lipton S.A., Ding S. (2011). Direct reprogramming of adult human fibroblasts to functional neurons under defined conditions. Cell Stem Cell.

[230] Abernathy D.G., Kim W.K., McCoy M.J., Lake A.M., Ouwenga R., Lee S.W., Xing X., Li D., Lee H.J., Heuckeroth R.O. (2017). MicroRNAs induce a permissive chromatin environment that enables neuronal subtype-specific reprogramming of adult human fibroblasts. Cell Stem Cell.

[231] Hu W., Qiu B., Guan W., Wang Q., Wang M., Li W., Gao L., Shen L., Huang Y., Xie G. (2015). Direct conversion of normal and Alzheimer’s disease human fibroblasts into neuronal cells by small molecules. Cell Stem Cell.

[232] Bhaduri A., Andrews M.G., Kriegstein A.R., Nowakowski T.J. (2020). Are organoids ready for prime time?. Cell Stem Cell.

[233] Hu J.L., Todhunter M.E., LaBarge M.A., Gartner Z.J. (2018). Opportunities for organoids as new models of aging. J. Cell Biol..

